# Self-supervised contrastive learning enables robust electrocardiogram-based cardiac classification

**DOI:** 10.1016/j.hroo.2026.01.016

**Published:** 2026-01-21

**Authors:** Deekshith Dade, Jake A. Bergquist, Rob S. MacLeod, Benjamin A. Steinberg, Tolga Tasdizen

**Affiliations:** 1 Scientific Computing and Imaging Institute, University of Utah, Salt Lake City, UT; 2 Department of Electrical & Computer Engineering, University of Utah, Salt Lake City, UT; 3 Nora Eccles Treadwell CVRTI, University of Utah, Salt Lake City, UT; 4 Department of Biomedical Engineering, University of Utah, Salt Lake City, UT; 5 Department of Medicine, Denver Health Medical Center, Denver, CO; 6 Department of Medicine, University of Colorado Anschutz Medical Campus, Aurora, CO

**Keywords:** Machine learning, Electrocardiogram, Self-supervised learning, Contrastive learning, Representational learning, Pre-training, Cardiac classification, Foundational models

## Abstract

**Background:**

Self-supervised contrastive learning has emerged as a powerful paradigm for learning generalizable representations from unlabeled data. In the context of electrocardiogram (ECG) analysis, such pre-training can significantly enhance classification performance, especially when labeled data is scarce.

**Objective:**

We aimed to investigate and improve contrastive self-supervised learning techniques for ECGs by systematically combining recent methodological advances in augmentation design, contrastive loss formulation, and encoder architectures.

**Methods:**

We implemented a contrastive pre-training framework combining vectorcardiography (VCG)-based physiologically-inspired augmentations, interlead, intersegment, contrastive loss, and patient-aware positive sampling. In addition, we developed a dual-stream architecture, extending the TemporalNet model by processing grouped ECG leads independently. Pretraining was conducted on a large corpus of approximately 1 million unlabeled ECGs. We evaluated performance on 2 downstream classification tasks—low left ventricular ejection fraction (LVEF) and high serum potassium chloride—using various levels of labeled supervision (1%, 5%, 10%, 50%, and 100%). The pre-trained models were compared with the randomly initialized models under both frozen and finetuned conditions.

**Results:**

Contrastive pre-training consistently improved performance across all supervision levels. In low-label settings (1%–10% supervision), the pre-trained model achieved 3%–4% higher area under the receiver operator curve on the LVEF task and 5%–7% higher area under the receiver operator curve on the potassium chloride task compared with the baseline. The performance gap narrowed with increased supervision but remained favorable toward pre-trained models.

**Conclusion:**

Our findings demonstrate that contrastive pre-training can substantially enhance ECG classification, especially when labeled data is limited. By unifying and extending ideas from recent literature into a scalable framework trained on 1 million ECGs, we provide practical guidance and architectural innovations for building strong ECG foundation models applicable to a broad range of clinical prediction tasks.

**Key Findings**

▪ **Self-supervised learning enables accurate electrocardiogram (ECG)-based cardiac classification even when labeled clinical data are scarce.** Models pretrained on unlabeled ECGs achieved substantially higher performance than standard supervised models when trained with limited labeled data, supporting use in real-world settings where expert annotations are expensive or unavailable.
▪ **ECG representations learned from large, unlabeled cohorts generalize across clinically distinct tasks.** Pretraining on 1 cardiac phenotype (eg, left ventricular dysfunction) improved performance on a different biochemical outcome (serum potassium chloride abnormality), suggesting that contrastive learning captures broadly relevant cardiac signal features rather than task-specific patterns.
▪ **Architectural inductive bias via lead grouping (LGTemporalNet) consistently enhances performance**, especially for the potassium chloride task, indicating that explicitly modeling inter-lead structure improves representation quality beyond standard temporal convolutional models.
▪ **Model adaptation to the target task remains important for clinical deployment.** Although pretrained models provided strong initial representations, fine-tuning on task-specific data yielded the highest performance, emphasizing the need for downstream calibration before clinical use.

## Introduction

The 12-lead electrocardiogram (ECG) is the most widely used and non-invasive diagnostic tool for assessing cardiac health.^1^ Clinical ECG interpretation allows characterization and diagnosis of a wide range of health problems, including cardiac arrhythmias, myocardial ischemia, electrolyte imbalances, structural abnormalities, and much more.^2^ As ECGs are often the first-line diagnostic tools in cardiac care and are routinely acquired in large volumes, they serve as a rich source of clinical insight and an ideal area for machine-learning-based approaches.

In recent years, there has been growing interest in automating ECG interpretation using machine learning (ML) techniques.^3^, ^4^, ^5^ Such automated analysis can facilitate real-time monitoring, reduce physician burden, and help in detecting subtle or rare patterns that may be overlooked in routine review. Studies have shown that deep-learning models trained on large, labeled ECG datasets can achieve expert-level performance in detecting specific conditions such as atrial fibrillation and long QT syndrome. They can also detect features not available for traditional clinical analysis, such as reduced ejection fraction.^3^ However, these successes are highly dependent on the availability of large, labeled datasets and disease-specific training, which limits generalizability and scalability.

The scarcity of high-quality, labeled data leads to challenges in clinical ECG ML that are difficult to overcome. Manual annotation of target outcomes is time-consuming (if available), and datasets often lack sufficient diversity to enable robust disease prediction. This scarcity has motivated efforts to explore representation learning approaches, specifically self-supervised learning (SSL) methods that allow models to learn general ECG features from unlabeled data.^6^, ^7^, ^8^ By decoupling the feature learning process from label availability, SSL holds promise for developing foundational ECG models that can be finetuned for multiple downstream tasks with minimal supervision. Such SSL approaches also allow for combining multiple, large, unlabeled datasets to leverage all available ECG data better and increase diversity and representation in the training process.

Among various SSL approaches, contrastive learning has emerged as a particularly effective strategy for learning discriminative and semantically meaningful representations from unlabeled data.^9^^,^^10^ Contrastive learning trains models to distinguish between similar (positive) and dissimilar (negative) examples by optimizing an embedding space in which similar inputs are clustered close together. For ECGs, this idea can be extended by defining “similarity” in terms of signal transformations or clinical attributes, for example, different leads from the same ECG, segments within a lead, or ECGs from the same patient. Importantly, the choice of positive and negative pairs, and the design of physiologically meaningful augmentations, play critical roles in learning useful representations.^11^

In this study, we present a contrastive SSL framework for ECG representation learning that integrates recent advances in augmentation design, patient-aware sampling, and model architecture. Our approach combines vectorcardiography (VCG) transformations, lead-based contrastive views, and co-training of lead-group encoders via a dual-stream model previously proposed for representation learning.^12^ We trained this system on a large dataset of approximately 1 million unlabeled ECGs to learn rich ECG embeddings without relying on diagnostic labels.^2^^,^^13^^,^^14^ To evaluate the effectiveness of our pre-training approach, we assessed its performance on 2 clinically important tasks: detection of low left ventricular ejection fraction (LVEF) and identification of abnormal serum potassium chloride (KCl) levels. We compared models initialized from scratch with those initialized using contrastive pre-training under varying levels of labeled supervision (1%, 5%, 10%, 50%, 100%). Across all experiments, pre-training led to consistent performance improvements, particularly in low-label settings, demonstrating the utility of SSL for scalable and label-efficient ECG analysis.

## Methods

This study was approved by the University of Utah Institutional Review Board. The requirement for informed consent was waived because of the retrospective nature of the study and minimal risk to participants. All procedures adhered to the principles outlined in the Declaration of Helsinki.

### Datasets and experimental setup

We utilized the following 3 datasets in this study: an LVEF-labeled ECG dataset (LVEF dataset), a large-scale unlabeled ECG dataset (1M dataset), and a serum potassium chloride-labeled ECG dataset (KCl dataset). These datasets were used across 2 primary experimental stages: (1) contrastive pre-training and (2) downstream classification. All data collection complied with the University of Utah’s internal review board requirements and all ECG samples from each subject were anonymized. Standard ECGs were recorded over 5 seconds at a 500 Hz sampling rate, and we only used the 8 independent leads (I, II, V1–V6), as the remaining standard leads are linear combinations of these signals. This choice reduced redundancy while preserving the full diagnostic information content of the 12-lead ECG and facilitates structured modeling of inter-lead relationships.

**LVEF dataset**. This dataset consisted of approximately 40,000 12-lead ECG recordings collected from the University of Utah Health system. Each ECG was matched with an LVEF value derived from echocardiography performed within 4 weeks. An echocardiographic LVEF <40% was the gold standard for ‘low LVEF’ (Table 1).

**Table 1:** Patient demographics in the LVEF *d*ataset. 19,355 patients matched of total 24,868 in this dataset

Variable	Count (percent)
Mean Age	55.61 years
Sex	
Men	10,297 (53.20)
Women	
American Indian and Alaska Native	332 (1.72)
Asian	390 (2.01)
Black or African American	407 (2.10)
Hispanic/Latino/a/x-other Hispanic/Latino/a/x	1274 (6.58)
Native Hawaiian and other Pacific Islander	263 (1.36)
Unknown	886 (4.58)
White or Caucasian	15,803 (81.65)
Ethnicity	
Hispanic/Latino	1574 (8.13)
Not Hispanic/Latino	16,985 (87.76)
Unknown	796 (4.11)
Atrial fibrillation	382 (1.97)
History of atrial fibrillation or flutter	477 (2.46)
Has ICD	2 (0.01)
Pacemaker	24 (0.12)
Diabetes	914 (4.72)
Ischemic heart disease	1032 (5.33)
Chronic heart failure	615 (3.18)
History of cerebrovascular disease	416 (2.15)
Hypertension	347 (1.79)
Chronic kidney disease	471 (2.43)
Valve disease	363 (1.88)

**Large-scale dataset**. We also curated a large-scale dataset of approximately 1 million ECG recordings from the same clinical archive (Table 2). These ECGs were unlabeled and used for contrastive pre-training. Recordings spanned a wide demographic and clinical range and were preprocessed to ensure lead completeness consistent sampling rates and segment duration. Additional statistics on the large-scale ECG pre-training dataset, including patient-level ECG distributions, are provided in the Supplemental Tables 1–3.

**Table 2:** Patient demographics in the large-scale *d*ataset. 195,425 patients matched of total 281,673 patients in this dataset (comprising 962,735 individual ECG recordings)

Variable	Count (percent)
Mean age	51.08 years
Sex	
Women	102,568 (52.48)
Race	
American Indian and Alaska Native	2877 (1.47)
Asian	4303 (2.20)
Black or African American	4380 (2.24)
Hispanic/Latino/a/x-other Hispanic/Latino/a/x	1016 (0.52)
Native Hawaiian and other Pacific Islander	156 (0.08)
Other	18,826 (9.63)
Other Pacific Islander	2344 (1.20)
Unknown	6372 (3.26)
White or Caucasian	155,151 (79.39)
Ethnicity	
Hispanic/Latino	21,647 (11.08)
Native Hawaiian/Other Pacific Islander	1 (0.00)
Not Hispanic/Latino	162,512 (83.16)
Unknown	11,265 (5.76)
Atrial fibrillation	5278 (2.70)
History of atrial fibrillation or flutter	5568 (2.85)
Has ICD	361 (0.18)
Pacemaker	884 (0.45)
Diabetes	22,089 (11.30)
Ischemic heart disease	9476 (4.85)
Chronic heart failure	5393 (2.76)
History of cerebrovascular disease	8743 (4.47)
Hypertension	28,452 (14.56)
Chronic kidney disease	7619 (3.90)
Valve disease	6753 (3.46)

**KCl dataset**. The potassium cohort of 59,000 8-lead ECGs was derived from the large-scale dataset by linking ECGs KCl measurements performed within a time window of 30 to 60 minutes (Table 3). ECGs with KCl values between 4.0–5.0 mEq/L were labeled as “normal,” while those with values >5.0 mEq/L were labeled as “high.” Low KCl values (<4.0 mEq/L) were excluded to create a binary classification problem focused on hyperkalemia detection.

**Table 3:** Patient demographics in the KCl Dataset. 29,695 patients of the total 59,944 in this dataset matched the inclusion criteria

Variable	Count (percent)
Mean age	54.97 years
Sex	
Men	16,427 (55.32)
Race	
American Indian and Alaska Native	455 (1.53)
Asian	517 (1.74)
Black or African American	631 (2.12)
Hispanic/Latino/a/x-other Hispanic/Latino/a/x	79 (0.27)
Native Hawaiian and other Pacific Islander	13 (0.04)
Other	2475 (8.33)
Other Pacific Islander	447 (1.51)
Unknown	718 (2.42)
White or Caucasian	24,360 (82.03)
Ethnicity	
Hispanic/Latino	2716 (9.15)
Not Hispanic/Latino	25,668 (86.44)
Unknown	1311 (4.41)
Atrial fibrillation	1112 (3.74)
History of atrial fibrillation or flutter	1172 (3.95)
Has ICD	75 (0.25)
Pacemaker	179 (0.60)
Diabetes mellitus	4887 (16.46)
Ischemic heart disease	2152 (7.25)
Chronic heart failure	1307 (4.40)
History of cerebrovascular disease	1419 (4.78)
Hypertension	6118 (20.60)
Chronic kidney disease	1983 (6.68)
Valve disease	1648 (5.55)

### Model architectures

We implemented 2 neural network architectures for ECG representation learning: the TemporalNet and a novel grouping-based extension referred to as Lead Grouping TemporalNet (LGTemporalNet). Both models are built to encode raw, multi-lead ECG signals into compact feature embeddings suitable for contrastive pre-training and downstream classification.

#### TemporalNet

**Figure 1:**
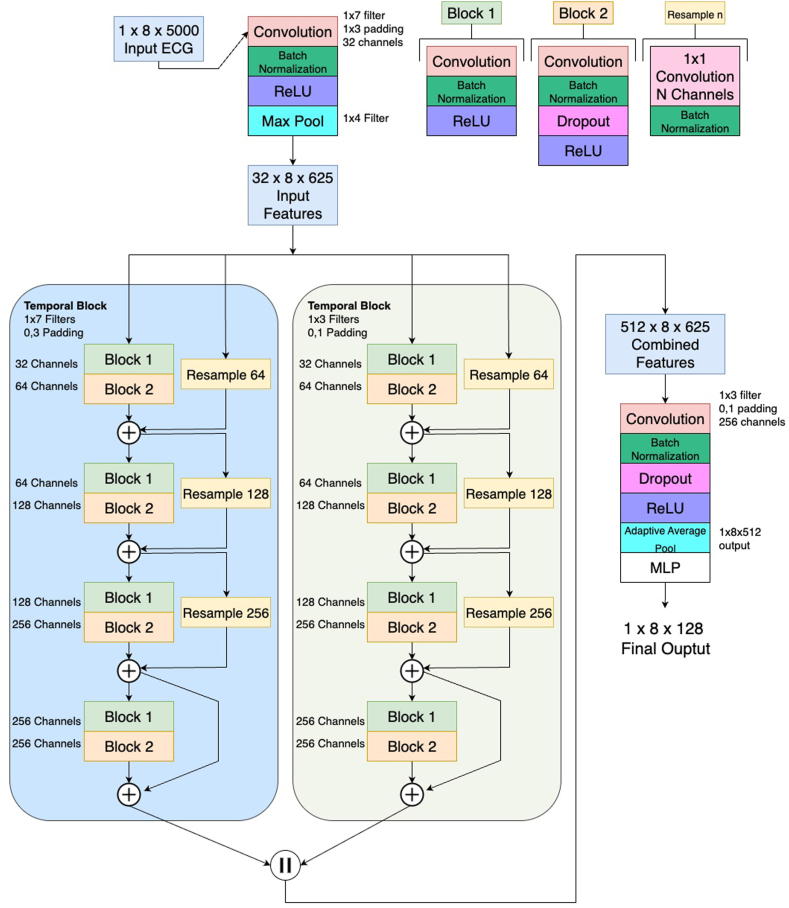
TemporalNet Architecture. Overview of the TemporalNet architecture for multi-lead ECG analysis. The model processes raw 1×8×5000 ECG signals through an initial convolution and pooling layer to extract temporal features. These features are then fed into 2 parallel temporal convolutional blocks, one with wide (1×7) and one with narrow (1×3) filters, to capture both long- and short-term dependencies. Each block is composed of residual sub-blocks with convolution, normalization, and dropout layers. The Resample operation uses a 1x1 convolution to align the number of channels in the residual connection. Features from both branches are concatenated, passed through additional convolution and pooling, and finally mapped to the output representation via a multilayer perceptron (MLP). The final output is a 1×8×128 representation suitable for downstream tasks. *ECG = electrocardiogram.*

TemporalNet^3^ (Figure 1) is a one-dimensional convolutional neural network designed to extract temporal features from each ECG lead independently. The architecture begins with a shared temporal convolutional backbone, composed of residual blocks and batch normalization, followed by temporal branches that separately process different aspects of the ECG signal. These branches are later integrated using channel-wise concatenation. The resulting features are pooled and projected into a lower-dimensional embedding space. When used in classification mode, a sigmoid layer is added on top of the embedding to output a binary prediction.

For contrastive pre-training, each of the 8 leads is processed independently and yields an embedding vector. Thus, for a single ECG, we obtain a multi-view representation comprising 8 embeddings of 128 dimensions per augmentation. These embeddings are normalized before passing into the contrastive loss function.

#### LGTemporalNet

**Figure 2:**
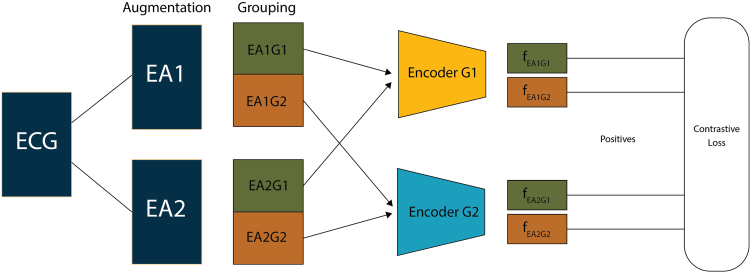
Contrastive pretraining with lead grouping and augmentations. Each ECG is augmented twice using a combination of VCG-based spatial transformations and temporal masking (EA1, EA2). The augmented signals are further split into 2 predefined lead groups (eg, EA1G1, EA1G2). Each lead group is passed through a dedicated encoder (Encoder G1, G2) to obtain embeddings. Pairwise contrastive loss is computed across all positive pairs from the same patient across augmentations and groups, while treating other patient embeddings as negatives. *ECG = electrocardiogram;* VCG *=* vectorcardiogram.

LGTemporalNet, as shown in Figure 2, is an extension of TemporalNet designed to explicitly model inter-lead relationships.^12^ Instead of processing all leads together, we partition the 8-lead ECG into 2 fixed lead groups (G1 and G2), each consisting of 4 leads. Each group is processed by a separate copy of the backbone encoder (eg, TemporalNet), resulting in embeddings per group. This structure is motivated by the spatial organization of ECG leads, which may capture complementary physiological information depending on anatomical site. For each ECG augmentation, this produces 2 embeddings; one from each group.

We employ 2 encoders in the LGTemporalNet architecture as a minimal multi-view design to explicitly model complementary ECG lead groupings as distinct but related modalities. This formulation aligns with prior multimodal contrastive learning approaches, in which paired encoders were used to learn shared representations across heterogeneous or partially overlapping views of the same underlying signal. 2-encoder designs have been shown to be effective in multimodal visual representation learning and in medical contrastive settings involving paired data, balancing effective representations with computational efficiency.^12^, ^15^, ^16^ Although extending the architecture to include additional encoders is possible, it would substantially increase memory and training cost without a clear theoretical guarantee of improved performance. The optimal number of encoders, therefore, remains an open question and is discussed as a limitation of the present study.

During contrastive training, LGTemporalNet yields 4 views per ECG (EA1-G1, EA1-G2, EA2-G1, EA2-G2). These views are included in the pairwise contrastive comparisons, allowing the model to learn invariances both across augmentations and across spatial partitions of the ECG.

**Figure 3:**
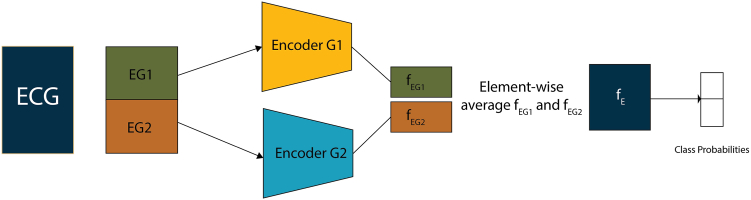
Downstream classification using grouped-lead encoders. During inference, each ECG is split into predefined lead groups (EG1, EG2), and each group is passed through its corresponding encoder (Encoder G1, G2), identical to those used during pre-training. The resulting embeddings $f_{EG1}$ and $f_{EG2}$ are averaged element-wise to form a joint representation $f_{r}$, which is passed to a classification head to predict the target label.

In classification mode (Figure 3), the 2 group-level embeddings were averaged and passed through a final linear + sigmoid layer to produce predictions. This setup enabled the model to leverage the distinct spatial patterns captured by the grouped encoders.

#### Embedding dimensions and design choices

In our experiments, we set the final embedding size to 128 for both architectures. For contrastive learning, the output embeddings were $L_{2}$-normalized prior to loss computation. Classification models used a frozen or finetuned version of the encoder, depending on the experimental setting. All architectures were trained using Adam optimizer,^17^ with weight clipping and cosine learning rate schedules applied during pre-training.

### Contrastive learning setup

We implemented a self-supervised contrastive learning framework for ECG pre-training by combining 3 key components: (1) physiologically-informed augmentations, (2) multi-view ECG representations at the lead level, and (3) patient-aware positive pair sampling.

**Figure 4:**
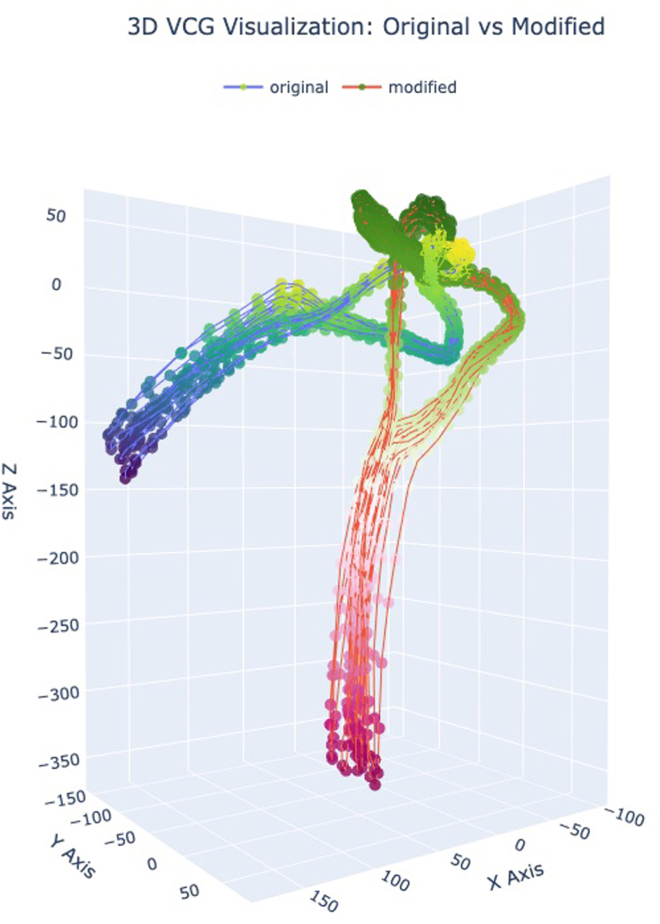
3D visualization of the vectorcardiogram (VCG) signal before and after transformation. The original trajectory is shown in blue, and the modified trajectory (after rotation and scaling) is shown in red. This visualization illustrates how geometric transformations affect the spatial morphology of the VCG loop.

1. **Physiologically-inspired augmentations (3KG).** We implemented the VCG augmentation strategy from the 3KG framework to simulate physiologically plausible signal variability.^2^ Each ECG was projected into a 3D vector space using the inverse Dower transform,^18^ followed by random 3D rotation and scaling. Specifically, we used a maximum rotation of $45^{\circ }$ and a scaling factor of up to 1.5×, which we found to produce stable and meaningful variability in lead morphology (Figure 4). The transformed signal was then projected back into ECG space using the Dower transform. We then applied temporal zero masking, where 50% of each lead’s signal was masked, mimicking signal dropout or electrode interference (Figure 5).

**Figure 5:**
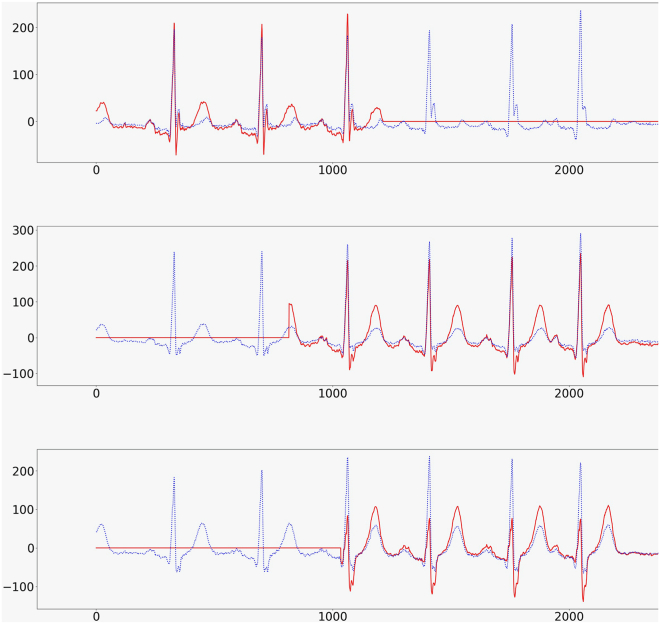
Example of pre-training input for masked ECG modeling. Blue trace denotes the original ECG signal, and the red overlay shows the augmented signal after transforming in VCG space and applying a 50% random mask.
2. **Multiview ECG representations.** Following the Contrastive Multi-Lead Coding formulation,^14^ each ECG lead was treated as an independent positive view of the same cardiac event. After augmenting an ECG, we computed a representation vector for each lead independently. The contrastive loss was then applied across all pairwise combinations of these lead embeddings. Unlike earlier approaches that average lead features before applying contrastive loss, Contrastive Multi-Lead Coding allowed the model to learn more fine-grained, lead-specific representations, while enforcing consistency across the multi-lead spatial context.
3. **Patient-aware positive sampling.** When multiple ECGs from the same patient were available, we treated them as additional positive pairs.^13^ This approach encouraged the model to learn patient-invariant features across temporal or morphological variations. Patient IDs were preserved and used during batch sampling to define valid positive pairs beyond augmentation.
4. **Contrastive loss function.** Each ECG in a batch underwent 2 independent augmentations, resulting in 2 augmented versions (EA1 and EA2). From each augmentation, we extracted one or more embeddings depending on the model:
  • **For TemporalNet**, each of the 8 ECG leads yields a separate embedding, producing a total of 2×8=16 views per sample.
  • **For LGTemporalNet**, each augmented ECG was split into 2 predefined lead groups (G1 and G2), and each group was passed through a separate encoder, resulting in 2(ECGs)×4(Leads)×2(Groups)=16 views per sample, of which 8 were from Encoder 1 and 8 from Encoder 2.

Let ${\left\{v_{i}\right\}}_{i=1}^{N}$ denote all view embeddings in a batch, where N depends on the batch size and the number of views per sample. We constructed a pairwise similarity matrix using cosine similarity between all embeddings. For each anchor embedding $v_{i}$, we defined the set of positives as all other embeddings derived from ECGs belonging to the same patient, regardless of which augmentation or lead (or group) they came from. All remaining embeddings in the batch were treated as negatives.

The contrastive objective encourages high similarity between all positive pairs and low similarity between negative pairs. This was implemented using a softmax-normalized exponential similarity:

$$l_{i}=-\frac{1}{\left|P\left(i\right)\right|}\sum_{j\in P\left(i\right)}\log\frac{\exp\left(\text{sim}\left(v_{i},v_{j}\right)/\tau \right)}{\sum_{k\neq i}\exp\left(\text{sim}\left(v_{i},v_{k}\right)/\tau \right)}$$

where P(i) is the set of positive indices for anchor i, $\text{sim}\left(v_{i},v_{j}\right)$ is the cosine similarity between embeddings, and τ is a temperature hyperparameter. The final loss is averaged across all anchors in the batch.

This formulation enables flexible and dense contrastive loss formulation: each anchor may have multiple positive pairs, arising from both augmentations and spatial transformations (eg, different leads or lead groups). It encourages invariance across time (augmentation), space (leads or lead groups), and patient-level variability, ensuring the model learns generalizable and robust ECG representations.

### Pretraining process

We performed contrastive pre-training independently using 2 datasets: the LVEF dataset and the large-scale ECG dataset.

**LVEF dataset pretraining.** The LVEF dataset was split into 90% of patients for pretraining and 10% for validation during classification. Each ECG was segmented into rhythm strips of 5 seconds (2500 samples), and 2 views were created via augmentation. We used spatial transformations in the VCG domain, specifically, random 3D rotations (up to $45^{\circ }$) and scaling (factor range of 1.5×) inspired by the 3KG approach, and temporal masking through zero-masking. The augmented views were processed as positive pairs for contrastive learning. Each batch was constructed using a set of 512 ECGs. Training was performed over 210 epochs with a learning rate of $1\times 10^{-4}$ using the Adam optimizer. A cosine annealing learning rate scheduler was applied, with a warm-up phase during the first 50 epochs.

**1M dataset pretraining.** For the 1M dataset, ECGs were filtered to ensure appropriate temporal alignment with clinical lab values (eg, potassium), retaining only valid and sufficiently long recordings. Each ECG was cropped or padded to 5 seconds and augmented similarly using VCG-space spatial transformations and zero masking. We again used a batch size of 512 and trained for 210 epochs using the Adam optimizer and a cosine annealing schedule with 50 warm-up epochs.

**Architectures**. 2 contrastive architectures were used: the TemporalNet, and the Lead Grouping variant of TemporalNet. The LGTemporalNet setup involved splitting ECG leads into 2 semantically coherent groups: group 1 contained leads I, II, V5, and V6, whereas group 2 contained leads V1, V2, V3, and V4 based on the correlation of prediction between leads.^3^ Each group was processed by an independent encoder during pre-training. For both models, representations were L2 normalized prior to loss computation.

**Contrastive optimization**. Contrastive training used a temperature-scaled loss with τ=0.1. The similarity between different lead-wise or augmentation-wise embeddings was computed for all combinations within a batch. Embeddings from the same patient, regardless of view or lead group, were treated as positives, whereas all others served as negatives. Gradients were clipped to a maximum norm of 1.0, and training was monitored using top-1 and top-5 accuracy from an auxiliary classification objective evaluated per batch.

### Finetuning and classification process

To evaluate the impact of self-supervised pre-training, we conducted comprehensive downstream classification experiments on 2 clinical tasks: predicting low LVEF from 4974 LVEF-labeled ECGs and high KCl from 5189 KCl-labeled ECGs. Our evaluation compared 3 training regimes across varying supervision levels.

• **Baseline:** The model was initialized with random weights and trained end-to-end from scratch on the target classification task.
• **Pretrained (Frozen):** The model was initialized with weights from the pre-training phase. All weights were frozen except for the final classification layer, which was trained on the task-specific data.
• **Pretrained (Finetuned):** The pre-trained weights were used as initialization. The model was then finetuned end-to-end, with a 2-tier learning rate: a higher learning rate for the final layer and a slower learning rate for the rest of the network.

We conducted all experiments using 5 different random seeds for robustness. For each seed, we split the signals into training and validation sets. To simulate varying levels of label availability, we finetuned models using 5 supervision levels: 1%, 5%, 10%, 50%, and 100% of the training data.

Each training configuration was run for a fixed number of epochs per supervision level, using Adam optimizers with a batch size of 512. For finetuning, we used a differential learning rate strategy, applying a higher rate (eg, $2\times 10^{-2}$) to the classification layer and a slower rate (eg, $1\times 10^{-4}$) to the pretrained backbone. The number of epochs per supervision level was adjusted to account for data size, with longer training on lower supervision settings.

### Model explainability via Grad-CAM

To provide insight into model decision-making, we performed post hoc explainability analysis using Gradient-weighted Class Activation Mapping (Grad-CAM), available in Supplemental Materials (Supplemental Figures 1–2). Grad-CAM was applied to the final convolutional layers of the baseline, pretrained-frozen, and pretrained-finetuned models to generate temporal importance maps for each ECG lead. For qualitative analysis, we selected representative test cases in which the baseline model produced incorrect predictions that were corrected by the pretrained models, including false negative to true positive and false positive to true negative transitions. Grad-CAM heatmaps were overlaid on the corresponding ECG waveforms to visualize regions contributing most strongly to the model predictions.

## Results

**Effectiveness of pretraining**. Across all experiments, we observed a consistent improvement in classification performance when models were initialized with contrastive pre-training compared with training from scratch. This improvement was particularly pronounced in low-supervision regimes (1%–10% of labeled data), highlighting the value of learning generalizable ECG representations. Both frozen and finetuned pretrained models outperformed the baseline, with finetuning typically offering the best performance. These trends held across both LVEF and KCl classification tasks and with different pretraining datasets (LVEF and 1M), reaffirming the utility of contrastive SSL for ECG-based diagnosis. Detailed per-seed performance metrics for each experiment, including all supervision levels and training configurations, are reported in the Supplemental Materials (Supplemental Tables 4–11).

### Pretraining the TemporalNet model using LVEF dataset

**Figure 6:**
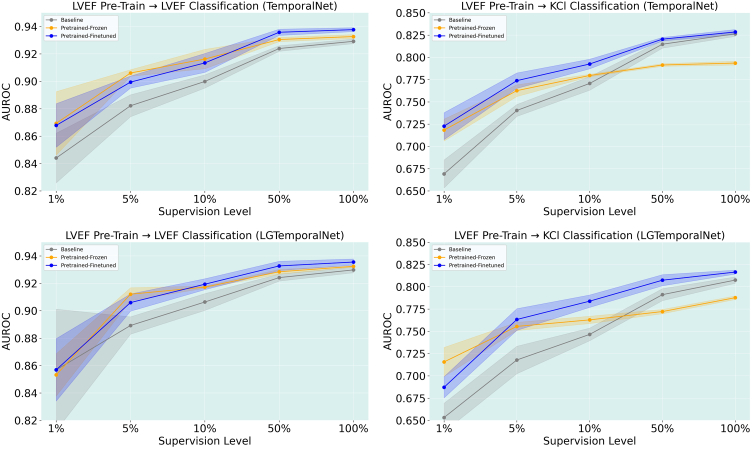
AUROC performance plots for LVEF and KC*l* classification tasks across different pretraining strategies and model architectures. Each row shows results from a different pretraining setting: (Top) LVEF dataset pre-training, (Bottom) LVEF dataset pre-training on LGTemporalNet, (Third) 1M dataset with standard TemporalNet, and (Bottom) 1M dataset with Grouping Leads TemporalNet. Results are shown separately for LVEF (left column) and KC*l* (right column) classification tasks.

**LVEF classification with LVEF pretraining.** Figure 6 illustrates the performance of models pre-trained on the LVEF dataset using the TemporalNet architecture and evaluated on the LVEF classification task. Contrastive pre-training resulted in a consistent improvement over the baseline across all levels of supervision. The largest gains were observed in low-data regimes, with the pre-trained-finetuned model outperforming the baseline by approximately 2.5% area under the receiver operator curve (AUROC) at the 1% and 5% supervision levels. As the amount of labeled data increased, the performance of all 3 model variants converged; however, the pre-trained-finetuned model maintained a consistent edge, achieving roughly 1% higher AUROC even at full supervision. It is important to note that in this setup, the same dataset (LVEF) was used for both pre-training and finetuning. As a result, the gains observed here may represent an upper bound on performance improvements because of contrastive pretraining, since the model is exposed to very similar data during both stages.

**KCl classification with LVEF pretraining.** Figure 6 presents results from the KCl classification task where the models were pre-trained on the LVEF dataset using the TemporalNet architecture. Similar to the LVEF classification setting, we observe that pretraining offered substantial improvements over the baseline, particularly under limited supervision. At 1% supervision, the pretrained-finetuned model improved AUROC by over 5% compared with the baseline. The gap narrowed at higher supervision levels, but the pretrained-finetuned model consistently maintained the highest performance. Interestingly, although the pretrained-frozen model showed early gains over the baseline, it plateaued more quickly and under-performed the finetuned variant at full supervision, likely because the LVEF pretraining learned features that were more immediately useful for the LVEF task, but less transferable to the KCl task. In this setting, finetuning allows for better adaptation of the learned representations to the new task, whereas frozen features provide limited benefit.

### LGTemporalNet model using the LVEF dataset

**LVEF classification with LVEF pretraining.** Figure 6 shows the performance of the LGTemporalNet architecture pre-trained on the LVEF dataset and evaluated on the LVEF classification task. Consistent with results observed for the TemporalNet architecture, contrastive pretraining improved performance across all supervision levels. The pretrained finetuned LGTemporalNet exhibited the strongest gains in low-data regimes, achieving approximately 3% higher AUROC than the baseline at 5% supervision. Although performance across all models converges as supervision increased, the pretrained finetuned variant maintained a modest but consistent advantage at higher supervision levels. Similar to the TemporalNet setting, this configuration represents a same dataset pretraining scenario, and thus the observed improvements likely reflected an upper bound on the benefits of contrastive pretraining when pretraining and finetuning distributions are closely aligned.

**KCl classification with LVEF pretraining.** Figure 6 presents results for KCl classification using the LGTemporalNet architecture pre-trained on the LVEF dataset. In this cross-task transfer setting, contrastive pre-training again yielded clear performance gains over the baseline, particularly under limited supervision. At 1% and 5% supervision, the pretrained-finetuned model outperformed the baseline by approximately 4%–5% AUROC, indicating that representations learned from LVEF data generalize effectively to potassium abnormality detection. As supervision increased, performance differences narrowed; however, the pretrained-finetuned LGTemporalNet consistently achieved the highest AUROC across all supervision levels. In contrast, the pretrained-frozen model demonstrated early improvements but plateaued at higher supervision levels, suggesting that task-specific finetuning is necessary to fully adapt LVEF-pretrained representations for the KCl task. These findings further support the role of finetuning in enabling effective cross-task transfer when the downstream classification objective differs from the pretraining task.

### Pretraining the TemporalNet model using 1M dataset

**Figure 7:**
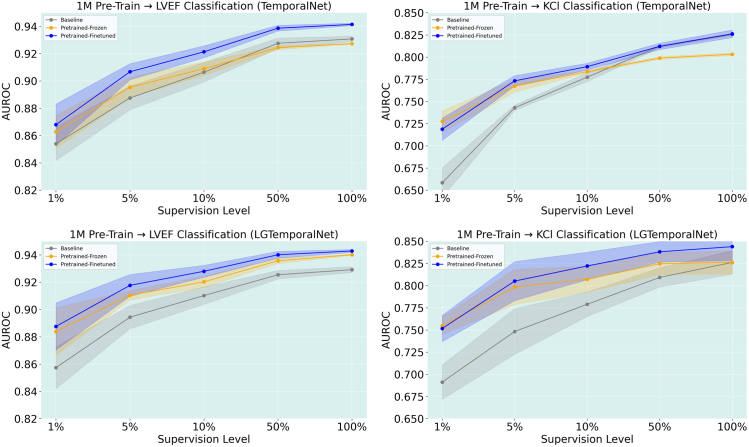
AUROC performance plots for LVEF and KC*l* classification tasks across different pretraining strategies and model architectures. Each row shows results from a different pretraining setting: (Top) 1M dataset with standard TemporalNet, and (Bottom) 1M dataset with LG TemporalNet. Results are shown separately for LVEF (left column) and KCl (right column) classification tasks.

**LVEF classification with 1M pretraining.** Figure 7 illustrates the performance of models pretrained on the 1M ECG dataset using the TemporalNet architecture and evaluated on the LVEF classification task. Compared with training from scratch, both pretrained variants showed clear improvements in AUROC across all levels of supervision, with the largest gains observed in the low-label regimes. For example, at 1% supervision, the pretrained-finetuned model outperformed the baseline by nearly 2%, and continued to maintain an edge of over 1% at full supervision. However, these improvements were not as pronounced as those observed when pretraining was performed on the LVEF dataset itself (Figure 7). Despite the broader scale and diversity of the 1M ECG dataset, it did not surpass the performance achieved when the model was pretrained and finetuned on LVEF data. This discrepancy can be attributed to the data reuse in the LVEF-pretrained setup. Specifically, there was overlap in the ECGs used for pre-training and finetuning, leading to a form of distributional alignment or even implicit data memorization. In contrast, the 1M-pretrained models were evaluated on entirely unseen downstream data from the LVEF task, representing a more realistic transfer scenario.

**KCl classification with 1M pretraining.** Figure 7 reports the KCl classification performance using models pre-trained on the 1M dataset. As with other settings, pre-training yielded substantial benefits in the low-supervision regime. At the 1% supervision level, the pre-trained-finetuned model outperformed the baseline by over 6% AUROC, and the frozen variant improved by nearly 7%, indicating strong generalization benefits from large-scale contrastive pre-training. These gains gradually declined with increasing supervision, with performance differences narrowing at 10% and largely converging at 50% and 100%. Notably, the pre-trained-frozen variant exhibited earlier saturation, whereas the finetuned model continued to outperform the baseline consistently across all levels.

When comparing this result with KCl classification models pretrained on the LVEF dataset, we observed that 1M pretraining delivered greater improvements in the low-data regime. Specifically, gains at 1% and 5% supervision were around 2%–3% higher for the 1M-pretrained models than for LVEF-pretrained models, suggesting that pretraining on a larger and more diverse dataset better equips the model to generalize under limited labeled supervision.

### Pretraining using 1M dataset with LGTemporalNet model

**LVEF classification with 1M pretraining and LGTemporalNet Model.** Figure 7 illustrates the LVEF classification performance using the Grouping Leads TemporalNet model pretrained on the 1M dataset. Across all supervision levels, both pretrained variants significantly outperformed the baseline. At 1% supervision, the pretrained-finetuned model achieved a 3.02% AUROC gain over the baseline, slightly higher than the 1M TemporalNet model without lead grouping (2.64%) and considerably stronger than the gains seen with LVEF pretraining (2.53%). This improvement persisted across all supervision levels, with the pretrained-finetuned model achieving a 1.37% gain at 100% supervision, outperforming the non-grouped model (1.06%) and LVEF-pretrained model (0.85%). These trends suggest that the inductive bias introduced by lead grouping may improve the model’s ability to extract discriminative representations from the ECG signal. Compared with the standard TemporalNet architecture, the grouped model exhibited both higher average performance and stronger gains relative to the baseline across nearly all settings. These results highlight the added value of integrating structured physiological priors into model design for ECG-based tasks.

**Figure 8:**
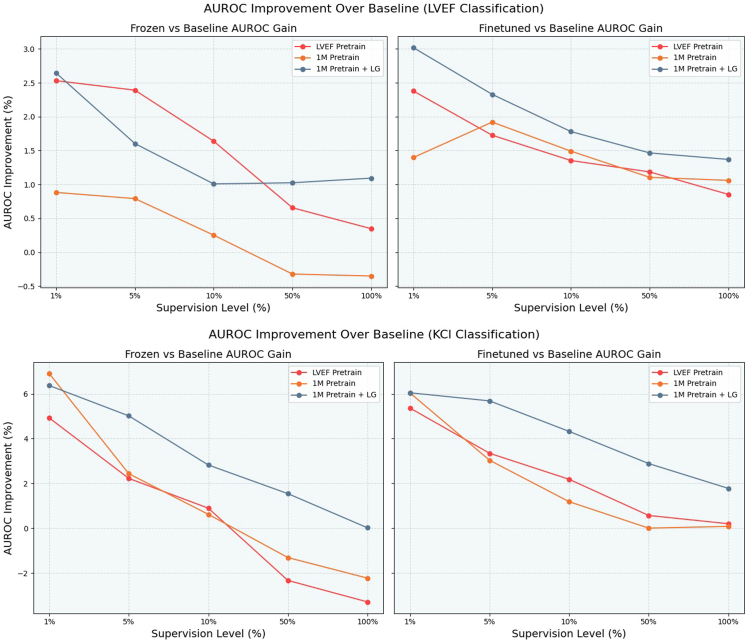
AUROC improvement over baseline for LVEF (top) and KCl (bottom) classification tasks across varying supervision levels (1%, 5%, 10%, 50%, *and* 100%). Each figure shows frozen (left) and finetuned (right) performance for 3 pretraining strategies: (i) pretraining on the LVEF dataset, (ii) pretraining on the 1M dataset, and (iii) pretraining on the 1M dataset with grouped-lead modeling (LGTemporalNet). Finetuned models consistently outperform frozen ones, and incorporating lead grouping leads to notably higher performance, especially in the KCl task.

**KCl classification with 1M pretraining and LGTemporalNet Model.** Figure 7 presents the results on the KCl classification task using the Grouping Leads TemporalNet model pretrained on the 1M dataset. The addition of lead grouping continued to yield notable improvements across supervision levels. At 1% supervision, the pretrained-finetuned model achieved a 6.05% AUROC gain over the baseline—–on par with the best performance seen in prior KCl experiments. As supervision increased, the advantage of grouped representations became even more pronounced: the finetuned model maintained a 5.7% gain at 5% supervision and over 4.3% at 10%, substantially outperforming the non-grouped counterpart. Even at 100% supervision, the grouped model retained a strong lead with a 1.78% improvement in AUROC (Figure 8). These findings suggest that combining contrastive pretraining with physiologically grounded lead-wise modeling offers robust benefits, especially in cross-task generalization scenarios like KCl classification.

**Comparative analysis of pretraining strategies.** Across all 6 experimental settings, contrastive pretraining consistently improved downstream classification performance over training from scratch. This trend held true across both tasks, LVEF and KCl, and for both model types, TemporalNet and LGTemporalNet. Notably, the benefit of pretraining was most pronounced under low-supervision conditions (1%–-10%), where pretrained models achieved up to 5%–6% AUROC improvement in some cases, underscoring the value of SSL in label-scarce environments (Figure 8).

**Qualitative explainability analysis.** Supplemental Section 1 and Figure 1, Figure 2 present Grad-CAM explanations and visualizations for representative LVEF and KCl classification cases, respectively. In both tasks, pretrained-frozen and pretrained-finetuned models demonstrated more structured and consistent temporal attention patterns compared with the baseline, particularly in cases where pretrained models corrected baseline misclassifications.

**Impact of pretraining dataset and model architecture.** Across all experiments, finetuned models consistently achieved the highest AUROC, outperforming both frozen variants and baseline models (Figure 8). Additional metrics including F1 Score, precision, sensitivity, and specificity are reported in the Supplemental Materials (Supplemental Figures 3–10). This trend was robust across both LVEF and KCl classification tasks and across all pretraining configurations. The advantage of finetuned models was particularly clear in low-supervision settings, where they demonstrated the largest improvements in AUROC over baselines.

When comparing pretraining datasets, we observed that models pretrained on the task-specific LVEF dataset performed competitively, even on the KCl task, especially under low supervision. Interestingly, pretraining on the much larger and more diverse 1M ECG dataset did not yield consistently superior results for KCl classification in the finetuned setting unless lead grouping was explicitly modeled. Only with the LGTemporalNet architecture did 1M pretraining significantly outperform LVEF pretraining in the KCl task.

Although frozen models also showed improvements over baseline in many cases, they plateaued quickly with increasing supervision and were often surpassed by both baseline and finetuned variants in the higher supervision regimes. This set of findings points to the limitations of using static, frozen representations and underscores the importance of downstream task adaptation through finetuning.

Overall, contrastive pretraining demonstrated consistent benefits across all supervision levels and tasks. pretrained models outperformed randomly initialized baselines in nearly every scenario, with the most substantial improvements occurring in low-supervision regimes. These findings highlight the power of SSL in enhancing ECG classification performance, particularly when paired with architectural inductive biases such as lead grouping.

## Discussion

Our results demonstrate the effectiveness of contrastive pre-training for ECG representation learning. Across all classification tasks and supervision levels, models initialized with self-supervised pre-training consistently outperformed those trained from scratch. The gains were especially pronounced in low-supervision regimes (1%–10% labeled data), affirming that contrastive learning can capture transferable cardiac signal representations. Notably, pretrained-finetuned models achieved the highest AUROCs in nearly every setting, whereas even the pretrained-frozen variants surpassed the baselines in most cases. These findings reinforce the growing body of evidence supporting the use of SSL in clinical domains.^19^

Beyond the general benefits of pretraining, our experiments highlight several nuanced trends. First, the advantage of contrastive learning was more pronounced in models trained on large, diverse datasets such as the 1M ECG corpus, enabling better generalization across both the LVEF and KCl classification tasks. Second, lead grouping emerged as a particularly effective architectural prior model using the LGTemporalNet consistently outperformed standard TemporalNet counterparts, especially in the KCl classification task where performance with 1M pre-training only matched LVEF pre-training in the finetuned case once lead grouping was introduced. This suggests that capturing spatial structure and inter-lead dependencies is important for downstream ECG discrimination, particularly in more heterogeneous or structurally complex tasks.

Our results show that the gains from pretraining decrease as more labeled data becomes available, but the performance margin never fully vanishes. Even at 100% supervision, finetuned pretrained models retain a 1%–2% AUROC edge in many scenarios, underscoring the lasting benefits of self-supervised initialization. These trends, visualized clearly in our comparative AUROC improvement plots (Figure 8), support a consistent pattern: contrastive pretraining is not just helpful under label scarcity, it provides a robust initialization strategy that enhances performance across datasets, and architectural choices.

Our findings have practical significance for clinical applications, where labeled ECG datasets are often limited, unavailable, and/or expensive to curate. The ability to learn robust representations from large unlabeled corpora like the 1M dataset enables the development of high-performing models using only a fraction of labeled data. This paradigm has the potential to democratize ECG-based artificial intelligence tools, allowing institutions with fewer annotations to still benefit from strong model performance. Additionally, the successful transfer of pretrained models to different classification tasks (eg*,* LVEF to KCl) underscores the generalizability of the learned embeddings, which is essential for real-world deployment across diverse diagnostic objectives.

Beyond pretraining strategies, our results validate the utility of architectural priors in model design. The lead grouping variant (LGTemporalNet) consistently outperformed its counterpart without grouping, particularly on the KCl classification task. This result suggests that incorporating domain-specific structural information—such as anatomical groupings of ECG leads—can further enhance representation learning.

Several prior studies have investigated self-supervised and contrastive learning approaches for ECG representation learning, demonstrating improved performance on tasks such as arrhythmia detection and general ECG classification. Our work builds directly on these efforts and can be viewed as a synthesis of ideas from multiple prior lines of research, combining large-scale contrastive pretraining, task transfer, and architectural inductive biases for ECG modeling. Consistent with earlier findings, these results confirm that contrastive objectives can extract meaningful and transferable cardiac representations from unlabeled data.^2^^,^^13^^,^^14^

Compared with earlier studies that often focused on a single downstream task, fixed supervision levels, or a specific architectural design, our study emphasizes systematic evaluation across a wide range of supervision regimes and examines how both pretraining dataset scale and model structure jointly influence transfer performance across heterogeneous tasks. Although multimodal SSL^20^ approaches have been shown to enhance representation learning in related domains, our study focused on single-modality ECG signals and instead evaluated robustness through extensive supervision scaling and architectural comparisons. These choices highlight both the strengths of contrastive ECG pretraining and important limitations of our current study, including restricted task diversity and the absence of explicit multimodal signals, which we identify as promising directions for future research.

### Limitations

This study has several limitations that should be acknowledged. One major limitation is the restricted scope of model architectures explored. We primarily relied on our TemporalNet model and its grouped-lead variant for all experiments. Although these architectures were intentionally designed to capture patterns in ECG signals, a more comprehensive comparison involving transformer-based models, recurrent networks, or hybrid approaches could reveal additional strengths and weaknesses of contrastive pre-training for ECGs.

Another limitation lies in the design of the lead grouping strategy. Although the 2-group configuration we adopted was motivated by prior literature and practical clinical considerations, it is only one of many possible grouping permutations. A broader search over grouping heuristics, potentially informed by lead correlations, anatomical positioning, or frequency characteristics, could uncover more optimal configurations and provide deeper insights into how grouping affects representation learning. Additionally, we did not perform fine-grained ablation studies on the Grouping Leads model to disentangle the benefits arising from grouping itself vs model capacity or input diversity.

Our experimental setup was focused on 2 downstream tasks: LVEF classification and KCl level classification. Although these tasks are clinically important and complementary, both were formulated as binary classification problems (reduced vs normal LVEF and elevated vs normal potassium), which simplifies clinically continuous variables into coarse categories. As a result, this evaluation does not capture the full range of cardiovascular pathologies or disease severity detectable via ECG. Extending our evaluation to multi-class or regression-based formulations, and to tasks such as arrhythmia detection, ischemia classification, or atrial fibrillation sub-typing, would provide a more comprehensive understanding of how well contrastively pretrained models generalize across diagnostic domains. Although AUROC is a widely accepted performance metric, exploring additional metrics such as sensitivity, specificity, or calibration error could shed light on practical deployment performance.

Finally, though our pretraining strategy incorporated 3KG-inspired spatial and temporal augmentations, the choice of transformations was relatively narrow. Other perturbations, such as amplitude scaling, temporal warping, or simulated noise artifacts, could further regularize model learning and enhance generalizability. Future studies could examine how these factors interact with pretraining to either improve or degrade downstream task accuracy, potentially guiding augmentation or data cleaning strategies.

## Conclusion

These findings open several promising directions for future research. First, the contrastive pre-training framework we developed can be applied to a broader spectrum of clinical classification tasks beyond LVEF and potassium levels. Particularly, it would be valuable to investigate its efficacy on underexplored or rare cardiac diseases that have serious clinical implications, for which annotated data is extremely limited. Demonstrating strong performance in these scenarios could unlock clinically useful tools for screening, early detection, and/or triage.

Second, collaboration with clinical experts is critical for evaluating the practical relevance of the learned representations. Although our models demonstrate strong performance on retrospective datasets, it remains important to validate whether their decisions align with physician expectations and domain knowledge. Feedback from cardiologists can help reveal potential biases, build trust in model outputs, and guide iterative model improvements toward clinical acceptance.

Finally, model interpretability remains a crucial challenge. For broader deployment, understanding which aspects of the ECG signal the model weights highly, particularly in the context of specific diseases, is essential. Tools for visualizing learned representations, highlighting salient regions of the waveform, or linking model features to known pathophysiological patterns can enhance explainability and facilitate clinician adoption. Developing explainable, pretrained ECG models that are both efficient and trustworthy will be a key objective for future research.

## Declaration of generative artificial intelligence (AI) and AI-assisted technologies in the manuscript preparation process

During the preparation of this work, the author(s) utilized ChatGPT-5 to enhance readability. After using this tool/service, the author(s) reviewed and edited the content as needed and take(s) full responsibility for the content of the published article.

## Disclosures

Benjamin A. Steinberg reports salary support from the National Institute of Health (NIH)/National Heart, Lung and Blood Institute (K23HL143156, R56HL168264, R21HL172288) and American Heart Association/Patient-Centered Outcomes Research Institute (18SFRN34110489), and other research support from Abbott, Cardiva, Sanofi, and AltaThera; and consulting to Sanofi, InCarda, Milestone, Pfizer, and AltaThera. Tolga Tasdizen reports salary support from NIH/National Heart, Lung and Blood Institute (R21HL172288). Rob MacLeod reports salary support from the Veteran Affairs Medical Center (5I01CX002758-02) and the NIH (R01HL174056-01 and 1R03NS145583). The remaining co-authors did not report any relevant disclosures.

## Footnotes

Appendix Supplementary data associated with this article can be found in the online version at https://doi.org/10.1016/j.hroo.2026.01.016.
