# Supplementary material for: Self-supervised contrastive learning enables robust electrocardiogram-based cardiac classification
Source: Heart Rhythm O2. 2026 Jan 21;7(4):757–70. doi: 10.1016/j.hroo.2026.01.016 (PMC13107047; doi:10.1016/j.hroo.2026.01.016)
Supplement: Supplementary Material [file mmc1.docx]

Supplemental Material

**Code and Pre-Trained Model:** https://github.com/Deekshith-Dade/ecg-contrastive

# Grad-CAM Analysis and Model Interpretability

To gain insight into the behavior of the proposed models, we performed a Grad-CAM analysis on representative test ECGs. Grad-CAM was applied to the final convolutional layers of the TemporalNet architectures to visualize temporal regions that most strongly influenced model predictions. This analysis was intended to provide qualitative evidence of how contrastive pretraining alters model attention compared to training from scratch. We focused specifically on clinically informative correction cases, selecting examples in which the baseline model produced incorrect predictions that were subsequently corrected by the pretrained models. Two types of transitions were examined: false negative to true positive (FN$\to$TP), highlighting recovery of missed pathology, and false positive to true negative (FP$\to$TN), highlighting suppression of spurious activations. This selection strategy ensures that the visualizations reflect meaningful behavioral differences between models rather than trivial agreement cases.

Figure S2 shows Grad-CAM visualizations for the KCL classification task, while Figure [1](#fig:gradcam_lvef_fn_tp) and Figure S1 presents corresponding examples for LVEF classification. For each example, Grad-CAM maps are shown for the baseline model, the pretrained-frozen model, and the pretrained-finetuned model across multiple ECG leads. Across both tasks, the baseline models exhibit diffuse and inconsistently distributed activation patterns, often highlighting regions outside of key cardiac events. In contrast, pretrained models, particularly the finetuned variants, demonstrate more structured and temporally aligned attention, frequently concentrating activation around physiologically salient waveform components such as QRS complexes. This shift in attention coincides with improved classification outcomes in the corrected cases.

Notably, pretrained-frozen models often show partial improvement over the baseline, indicating that contrastive pretraining alone learns useful representations. However, pretrained-finetuned models consistently exhibit the most coherent and discriminative activation patterns, suggesting that end-to-end finetuning further refines these representations to better align with task-specific clinical signals. While Grad-CAM does not provide a complete explanation of model reasoning, these qualitative results support the hypothesis that contrastive pretraining encourages the learning of more meaningful ECG representations. The observed alignment between improved predictions and focused temporal attention provides additional evidence that the performance gains reported in this study are associated with more physiologically relevant feature utilization rather than spurious correlations.

**
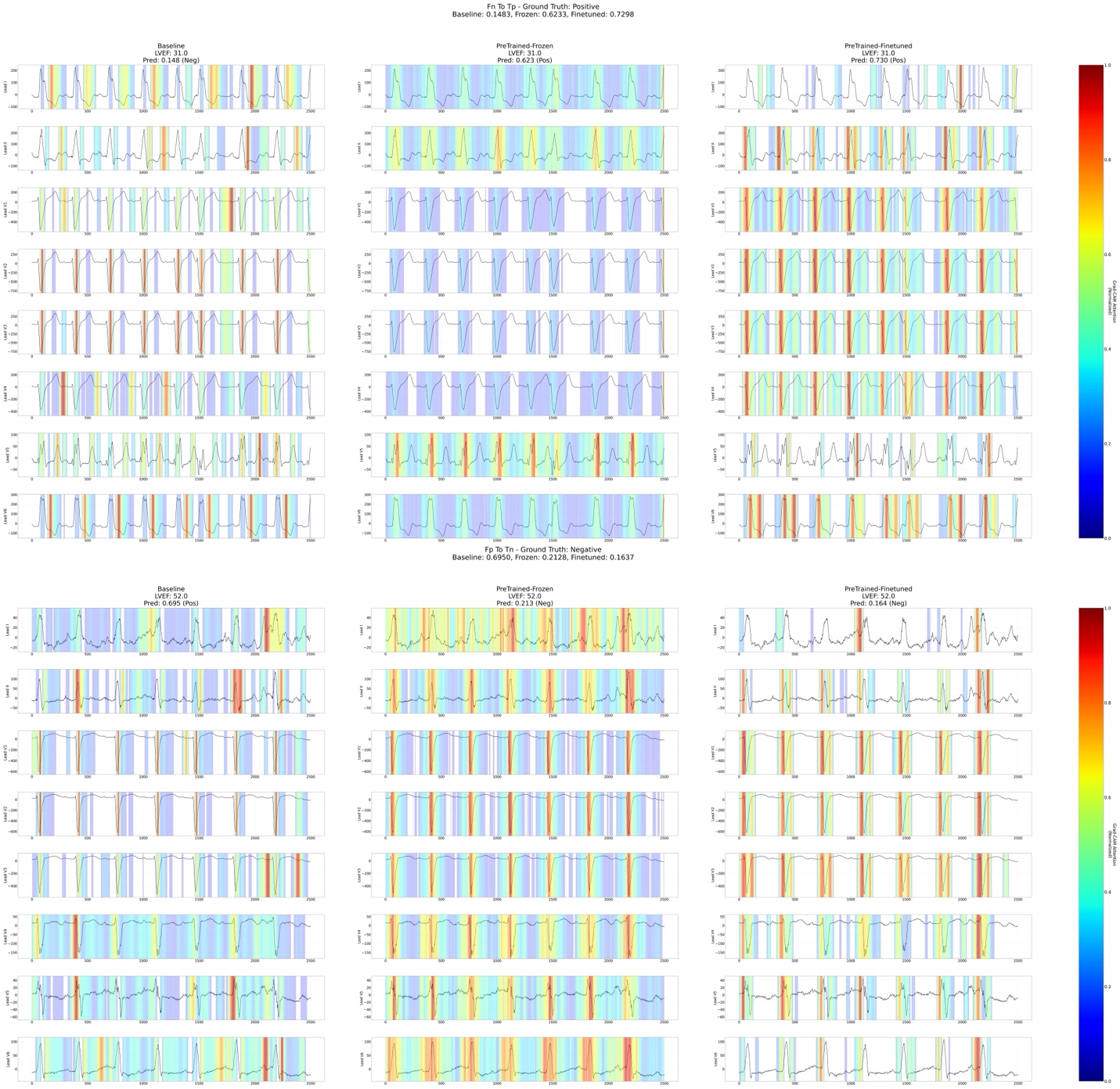
**

**FigureS1:** Grad-CAM visualizations for representative LVEF classification correction cases. Each subfigure compares baseline, pretrained-frozen, and pretrained-finetuned models across multiple ECG leads. Warmer colors indicate higher contribution to the model prediction. (Top) False Negative $\to$ True Positive correction for LVEF classification. (Bottom) False Positive $\to$ True Negative correction for LVEF classification.

**
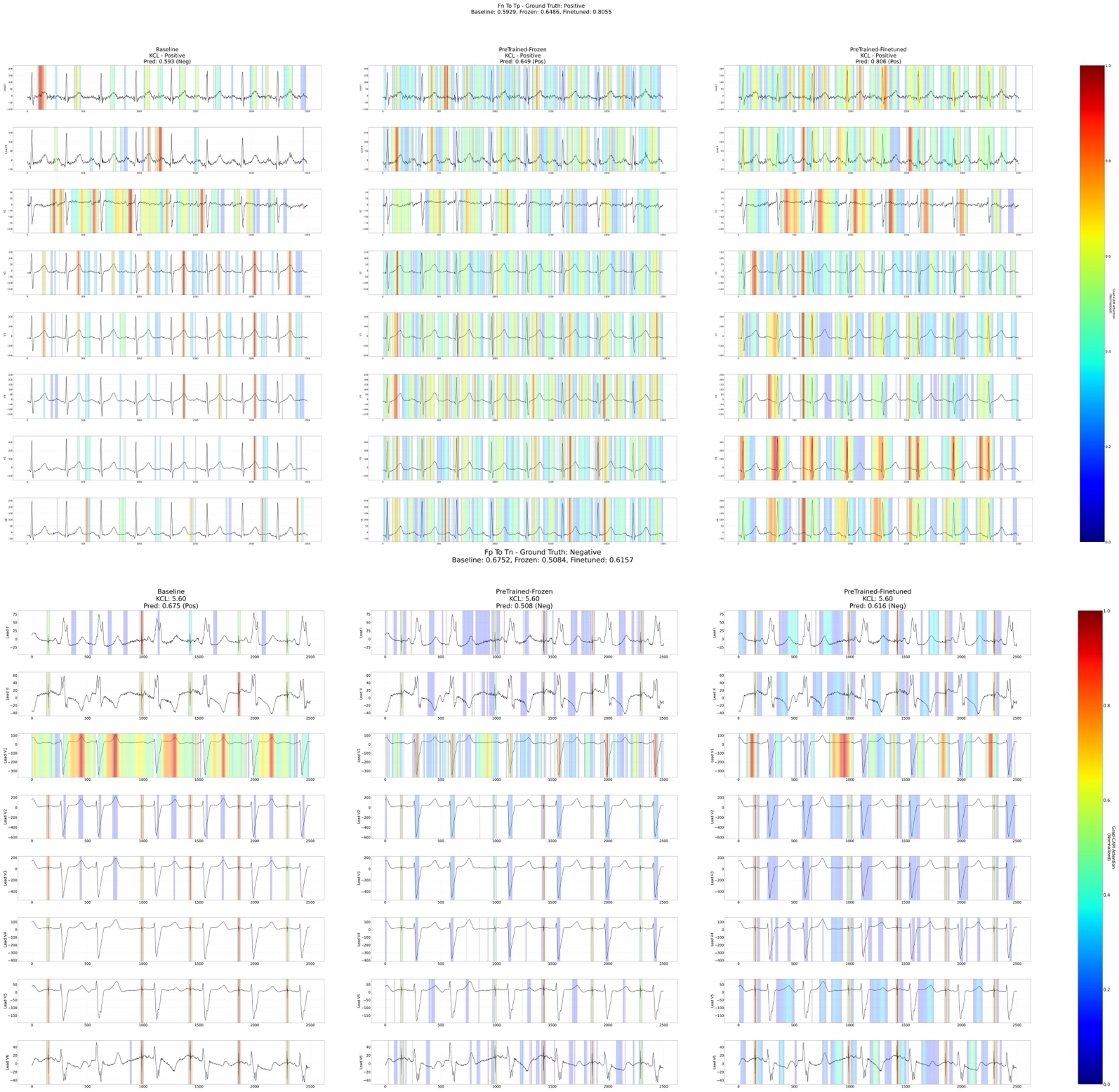
**

**FigureS2:** Grad-CAM visualizations for representative KCL classification correction cases. Baseline, pretrained-frozen, and pretrained-finetuned models are shown for identical ECG segments. (Top) False Negative $\to$ True Positive correction for KCL classification. (Bottom)False Positive $\to$ True Negative correction for KCL classification.


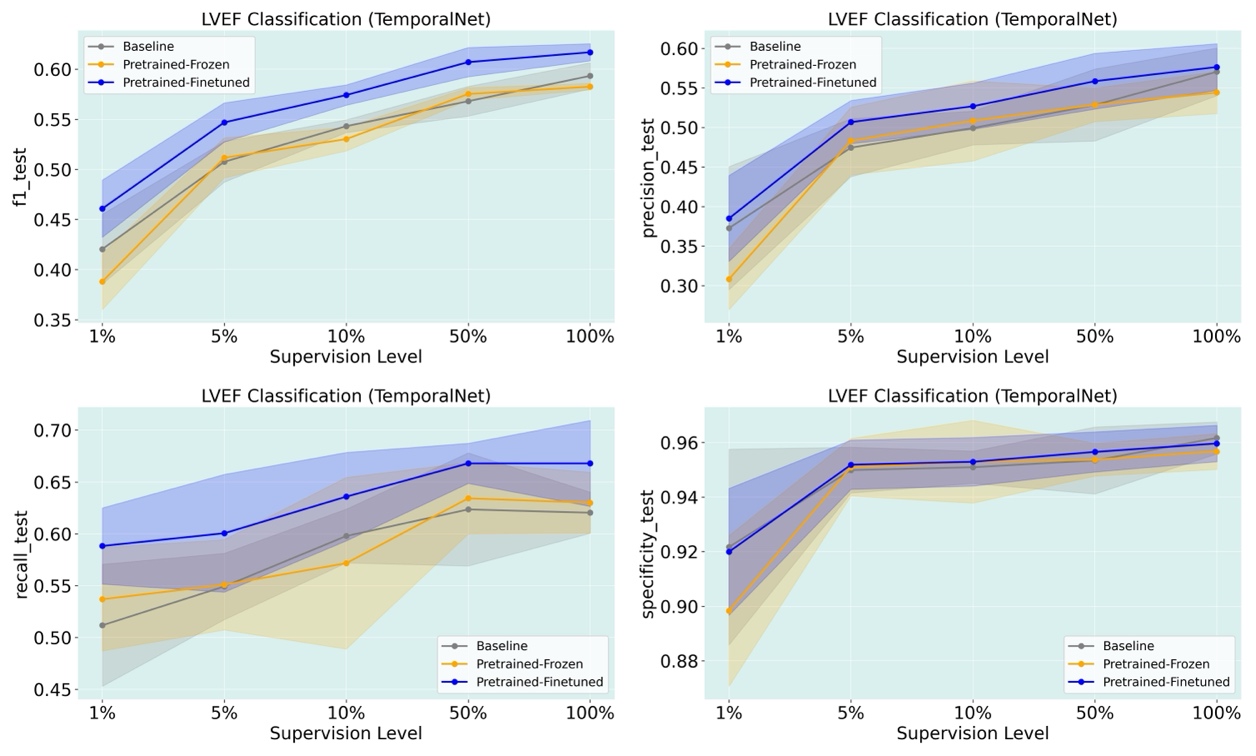


**FigureS3:** Additional performance metrics for LVEF classification using the TemporalNet architecture pretrained on LVEF data. Metrics include F1-score, precision, recall (sensitivity), and specificity across supervision levels. Shaded regions denote standard deviation across runs.

**
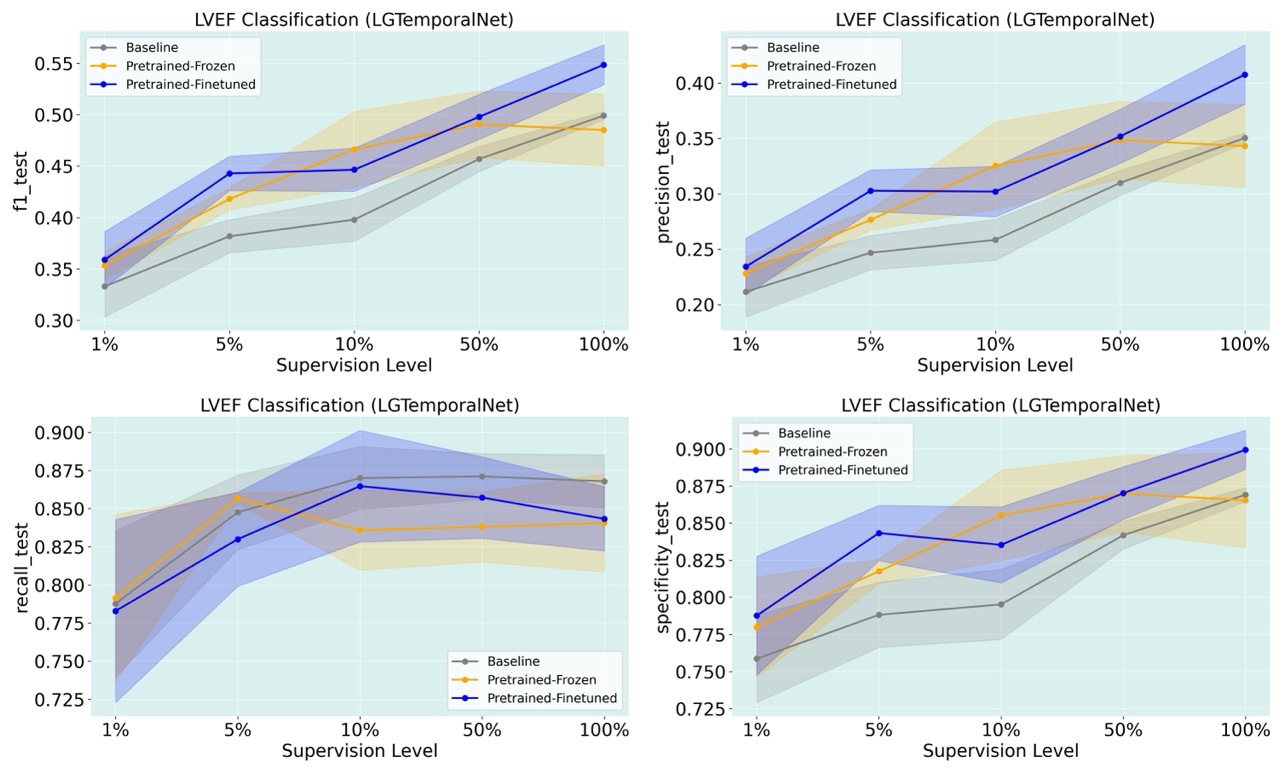
**

**FigureS4:** Additional performance metrics for LVEF classification using the Lead Grouping TemporalNet architecture pretrained on LVEF data. Metrics include F1-score, precision, recall (sensitivity), and specificity across supervision levels. Shaded regions denote standard deviation across runs.

**
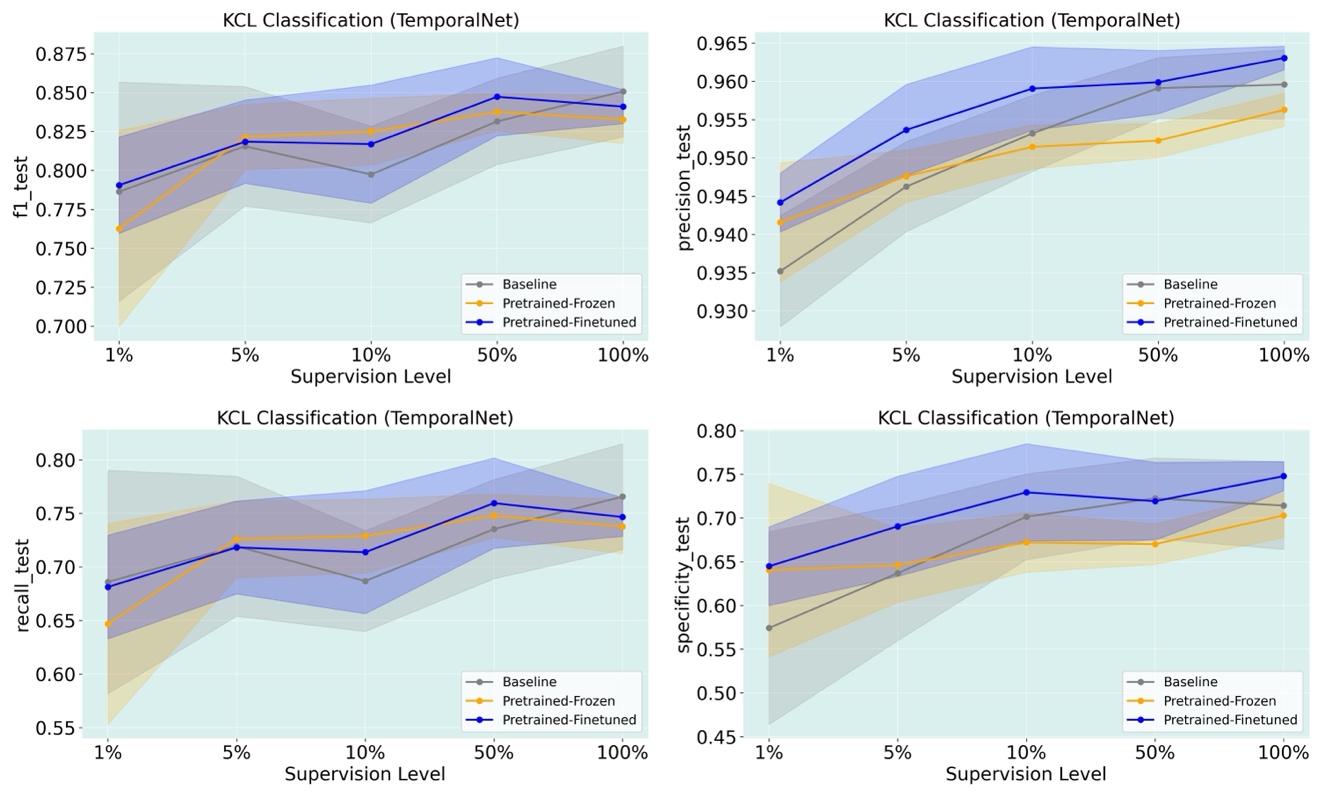
**

**FigureS5:** Additional performance metrics for KCL classification using the TemporalNet architecture pretrained on LVEF data. Metrics include F1-score, precision, recall (sensitivity), and specificity across supervision levels. Shaded regions denote standard deviation across runs.

**
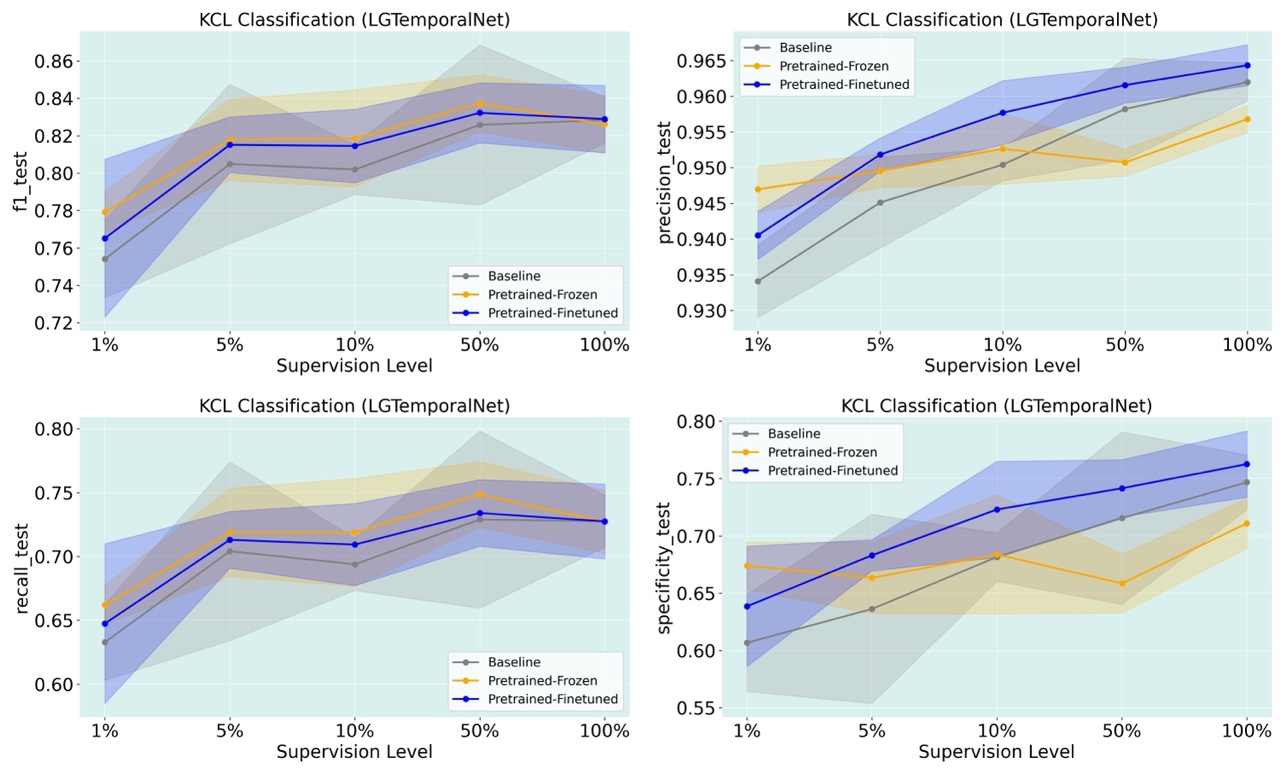
**

**FigureS6:** Additional performance metrics for KCL classification using the Lead Groupings TemporalNet architecture pretrained on LVEF data. Metrics include F1-score, precision, recall (sensitivity), and specificity across supervision levels. Shaded regions denote standard deviation across runs.

**
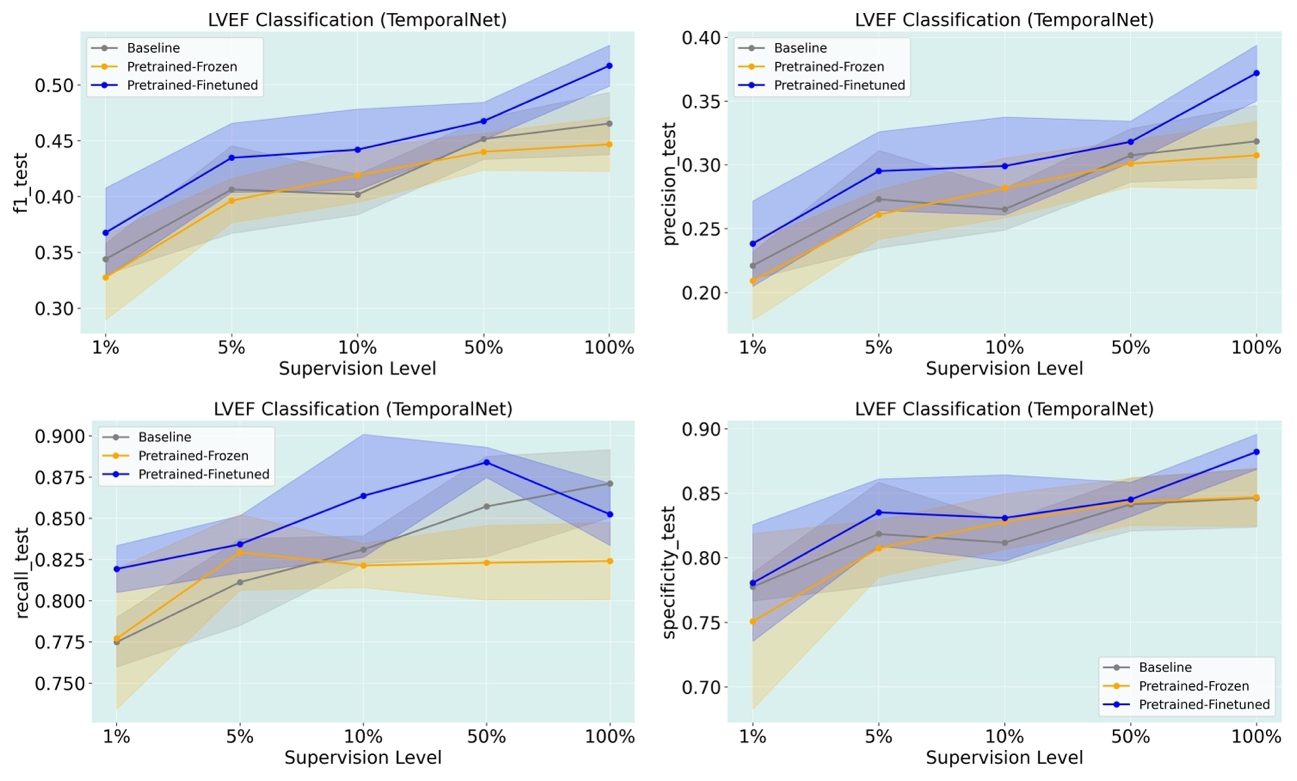
**

**FigureS7:** Additional performance metrics for LVEF classification using the TemporalNet architecture pretrained on 1M data. Metrics include F1-score, precision, recall (sensitivity), and specificity across supervision levels. Shaded regions denote standard deviation across runs.

**
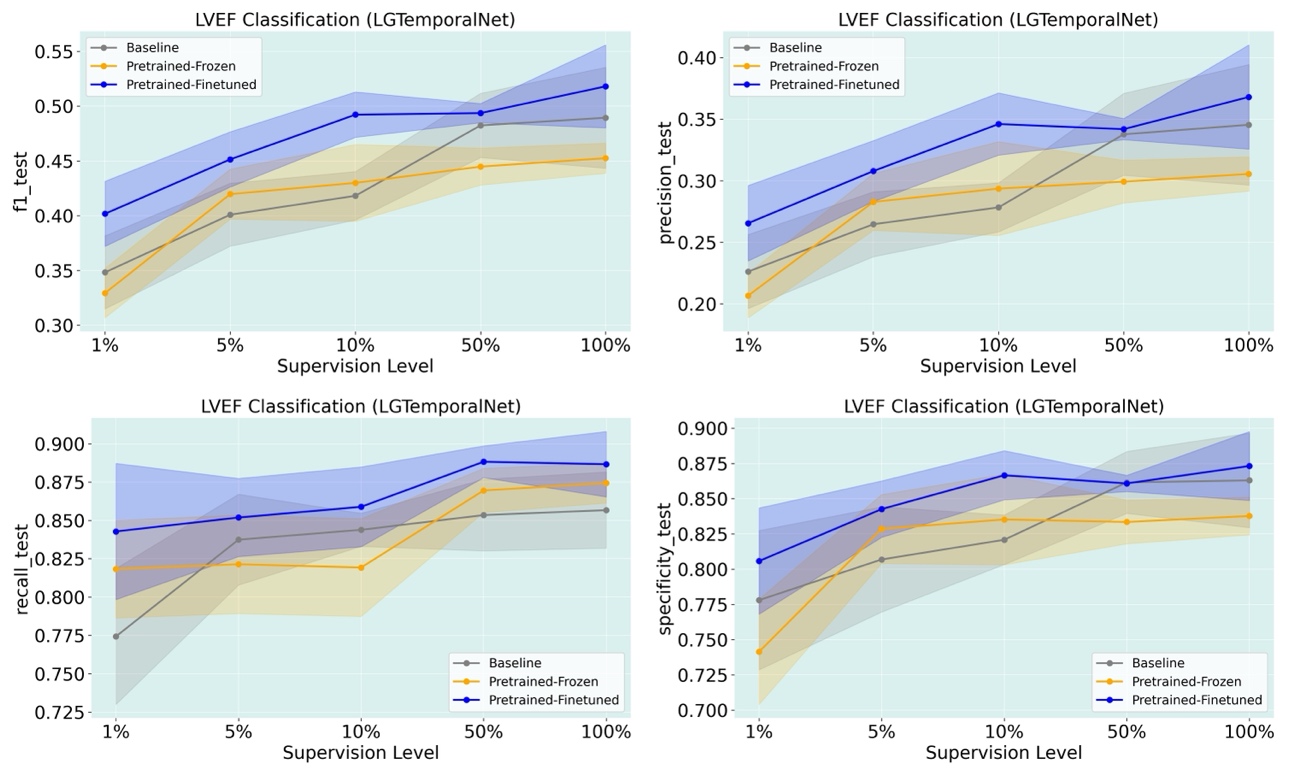
**

**FigureS8:** Additional performance metrics for LVEF classification using the Lead Grouping TemporalNet architecture pretrained on 1M data. Metrics include F1-score, precision, recall (sensitivity), and specificity across supervision levels. Shaded regions denote standard deviation across runs.

**
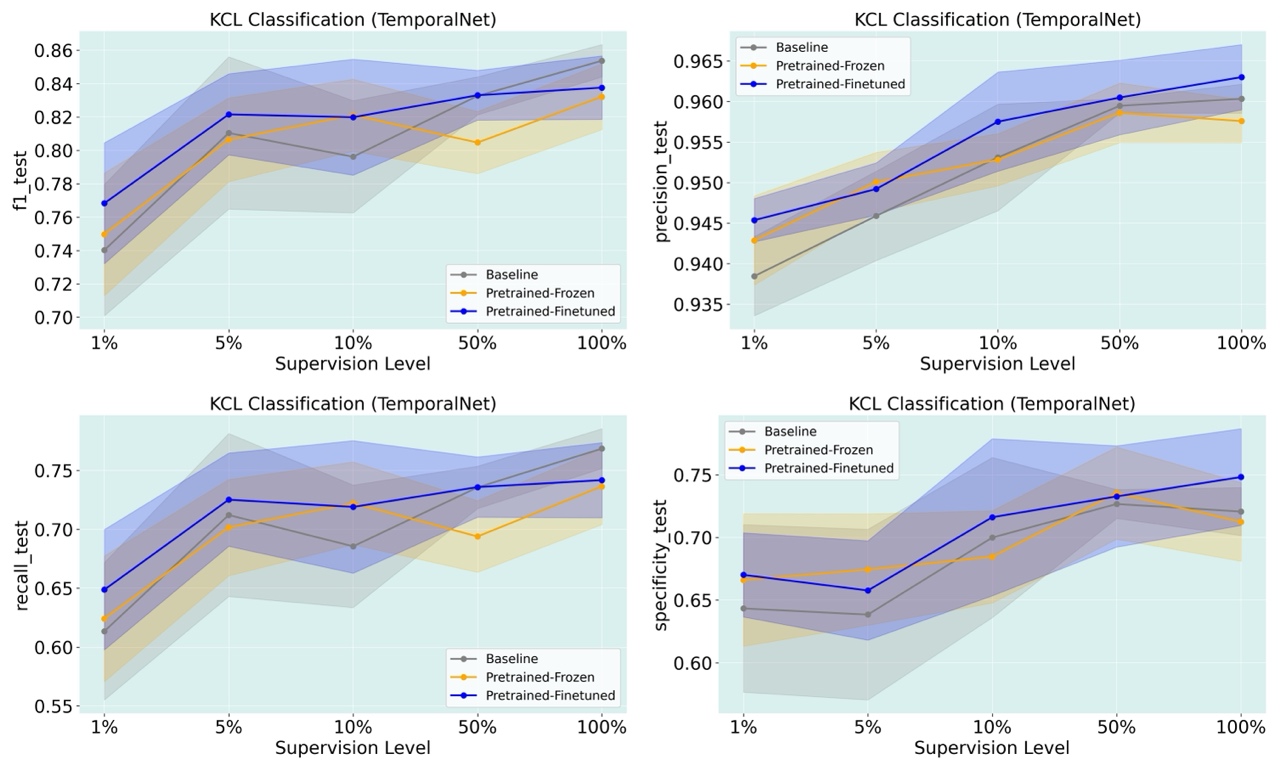
**

**FigureS9:** Additional performance metrics for KCL classification using the TemporalNet architecture pretrained on 1M data. Metrics include F1-score, precision, recall (sensitivity), and specificity across supervision levels. Shaded regions denote standard deviation across runs.

**
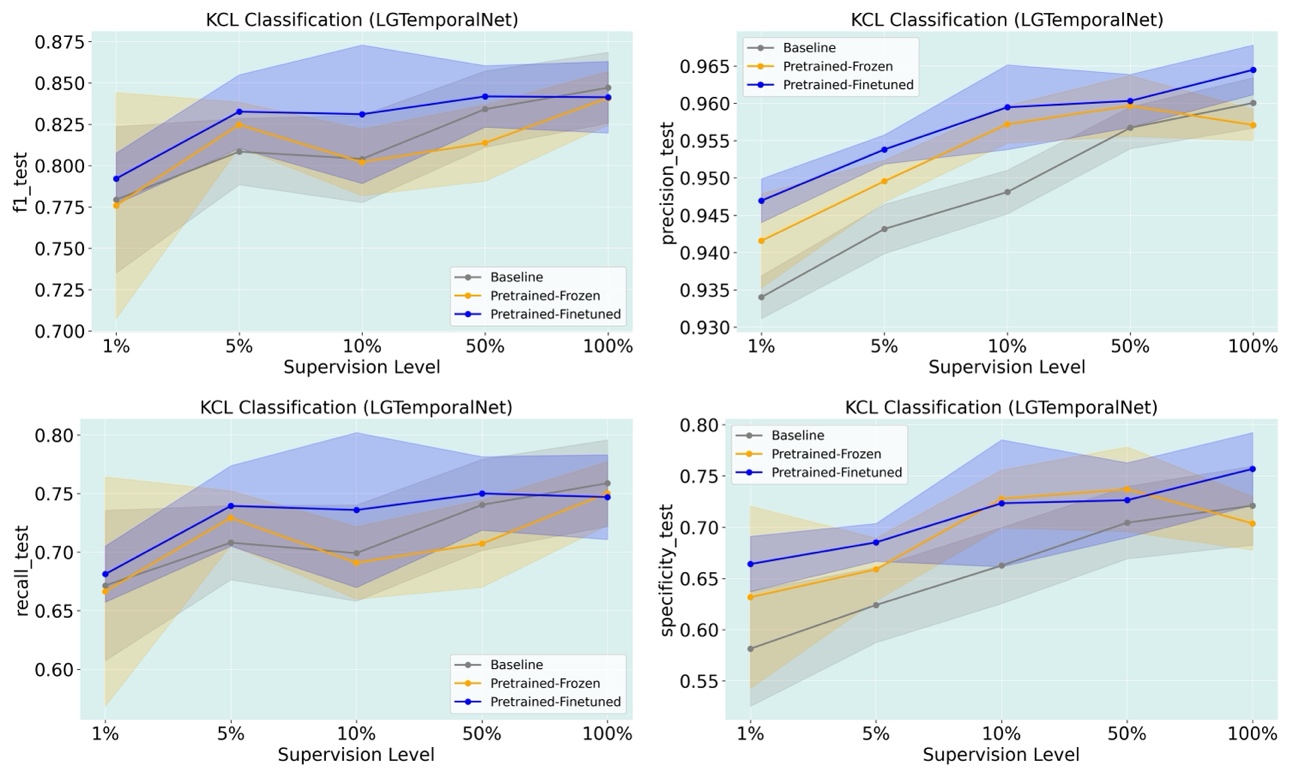
**

**FigureS10:** Additional performance metrics for KCL classification using the Lead Grouping TemporalNet architecture pretrained on 1M data. Metrics include F1-score, precision, recall (sensitivity), and specificity across supervision levels. Shaded regions denote standard deviation across runs.

# Large-Scale ECG Dataset Statistics

**TableS1:** Overview of the 1M ECG Pretraining Dataset

| **Statistic** | **Value** |
| --- | --- |
| Total ECGs | 962,735 |
| Unique patients | 281,673 |
| Average ECGs per patient | 3.42 |
| Median ECGs per patient | 2 |
| Minimum ECGs per patient | 1 |
| Maximum ECGs per patient | 470 |

**TableS2:** Distribution of ECG Counts per Patient

| **Statistic** | **Value** |
| --- | --- |
| Mean | 3.42 |
| Standard deviation | 5.91 |
| 25th percentile (Q1) | 1 |
| 50th percentile (Median) | 2 |
| 75th percentile (Q3) | 3 |
| 90th percentile | 7 |
| 95th percentile | 12 |
| 99th percentile | 27 |

**TableS3:** Patient-Level Contribution to the 1M ECG Dataset

| **Patient Category** | **Patients** | **ECGs Contributed** |
| --- | --- | --- |
| 1 ECG | 133,376 | 133,376 |
| 2–5 ECGs | 106,607 | 306,899 |
| 6–10 ECGs | 25,116 | 188,405 |
| 11–20 ECGs | 11,602 | 164,535 |
| 21–50 ECGs | 4,461 | 129,934 |
| 51–100 ECGs | 441 | 28,470 |
| 100+ ECGs | 70 | 11,116 |

The large-scale ECG dataset used for contrastive pretraining consists of 962,735 ECGs from 281,673 unique patients. While nearly half of patients contribute a single ECG, over 50% of patients have multiple ECG recordings, enabling patient-aware positive sampling during contrastive learning. A small fraction of patients contribute a large number of ECGs.

## Table S.4: AUROC results across five random seeds for LVEF Pretraining, LVEF Classification Task (TemporalNet)

| **Seed** | **446 ECGs Baseline** | **446 ECGs Frozen** | **446 ECGs Finetuned** | **2,238 ECGs Baseline** | **2,238 ECGs Frozen** | **2,238 ECGs Finetuned** | **4,476 ECGs Baseline** | **4,476 ECGs Frozen** | **4,476 ECGs Finetuned** | **22,380 ECGs Baseline** | **22,380 ECGs Frozen** | **22,380 ECGs Finetuned** | **44,762 ECGs Baseline** | **44,762 ECGs Frozen** | **44,762 ECGs Finetuned** |
| --- | --- | --- | --- | --- | --- | --- | --- | --- | --- | --- | --- | --- | --- | --- | --- |
| 42 | 84.91% | 88.36% | 88.62% | 88.11% | 90.37% | 89.20% | 90.09% | 91.82% | 91.48% | 92.38% | 93.05% | 93.96% | 92.91% | 93.24% | 93.90% |
| 43 | 84.55% | 83.94% | 87.66% | 87.08% | 90.68% | 89.88% | 89.24% | 91.00% | 90.05% | 92.42% | 93.17% | 93.53% | 93.06% | 93.26% | 93.50% |
| 44 | 81.41% | 84.54% | 83.89% | 87.81% | 90.29% | 89.86% | 90.75% | 92.53% | 92.00% | 92.46% | 93.26% | 93.53% | 93.01% | 93.30% | 93.77% |
| 45 | 84.09% | 87.88% | 86.66% | 89.46% | 90.87% | 90.41% | 89.97% | 92.15% | 91.72% | 92.04% | 92.95% | 93.62% | 92.60% | 93.04% | 93.88% |
| 46 | 87.07% | 89.96% | 87.07% | 88.62% | 90.83% | 90.36% | 89.90% | 90.64% | 91.38% | 92.67% | 92.82% | 93.25% | 93.02% | 93.50% | 93.81% |

## Table S.5: AUROC results for KCL classification pretrained on LVEF Data (TemporalNet)

| **Seed** | **523+47 Baseline** | **523+47 Frozen** | **523+47 Finetuned** | **2,618+237 Baseline** | **2,618+237 Frozen** | **2,618+237 Finetuned** | **5,236+474 Baseline** | **5,236+474 Frozen** | **5,236+474 Finetuned** | **26,183+2,371 Baseline** | **26,183+2,371 Frozen** | **26,183+2,371 Finetuned** | **52,367+4,742 Baseline** | **52,367+4,742 Frozen** | **52,367+4,742 Finetuned** |
| --- | --- | --- | --- | --- | --- | --- | --- | --- | --- | --- | --- | --- | --- | --- | --- |
| 42 | 67.80% | 72.33% | 72.24% | 74.47% | 75.37% | 77.23% | 76.82% | 77.75% | 79.50% | 81.93% | 79.21% | 81.86% | 82.75% | 79.73% | 83.24% |
| 43 | 68.89% | 72.51% | 73.66% | 74.47% | 77.11% | 78.78% | 77.60% | 78.04% | 79.92% | 82.24% | 80.82% | 79.29% | 82.79% | 79.27% | 82.51% |
| 44 | 64.75% | 69.73% | 69.82% | 72.89% | 76.02% | 76.40% | 75.95% | 78.10% | 79.13% | 81.33% | 78.89% | 81.83% | 79.22% | 82.16% | 82.65% |
| 45 | 66.21% | 72.79% | 73.38% | 74.32% | 76.46% | 77.13% | 77.92% | 77.95% | 78.45% | 81.82% | 79.19% | 82.25% | 82.88% | 79.19% | 82.97% |

## Table S.6: AUROC results for LVEF classification using Lead Groupings TemporalNet (LVEF Pretraining)

| **Seed** | **446 ECGs Baseline** | **446 ECGs Frozen** | **446 ECGs Finetuned** | **2,238 ECGs Baseline** | **2,238 ECGs Frozen** | **2,238 ECGs Finetuned** | **4,476 ECGs Baseline** | **4,476 ECGs Frozen** | **4,476 ECGs Finetuned** | **22,380 ECGs Baseline** | **22,380 ECGs Frozen** | **22,380 ECGs Finetuned** | **44,762 ECGs Baseline** | **44,762 ECGs Frozen** | **44,762 ECGs Finetuned** |
| --- | --- | --- | --- | --- | --- | --- | --- | --- | --- | --- | --- | --- | --- | --- | --- |
| 42 | 84.830% | 87.392% | 88.963% | 88.610% | 90.752% | 89.912% | 91.142% | 91.824% | 92.037% | 92.797% | 93.000% | 93.764% | 93.125% | 93.252% | 93.500% |
| 43 | 86.153% | 83.611% | 85.035% | 89.125% | 91.588% | 89.812% | 89.619% | 91.700% | 91.246% | 92.220% | 92.782% | 93.412% | 92.750% | 93.269% | 93.447% |
| 44 | 79.500% | 85.272% | 82.078% | 88.273% | 90.578% | 90.811% | 90.283% | 91.687% | 92.409% | 92.622% | 92.966% | 93.386% | 92.822% | 93.258% | 93.485% |
| 45 | 84.495% | 86.690% | 85.407% | 90.067% | 91.333% | 91.077% | 91.273% | 91.727% | 92.197% | 92.355% | 92.943% | 92.778% | 92.898% | 93.284% | 93.350% |
| 46 | 93.350% | 83.640% | 86.978% | 88.555% | 91.798% | 91.382% | 90.878% | 91.598% | 91.803% | 92.110% | 92.536% | 93.020% | 93.317% | 93.138% | 93.995% |

## Table S.7: AUROC results for KCL classification using Grouped Leads TemporalNet (LVEF Pretraining)

| **Seed** | **523+47 Baseline** | **523+47 Frozen** | **523+47 Finetuned** | **2,618+237 Baseline** | **2,618+237 Frozen** | **2,618+237 Finetuned** | **5,236+474 Baseline** | **5,236+474 Frozen** | **5,236+474 Finetuned** | **26,183+2,371 Baseline** | **26,183+2,371 Frozen** | **26,183+2,371 Finetuned** | **52,367+4,742 Baseline** | **52,367+4,742 Frozen** | **52,367+4,742 Finetuned** |
| --- | --- | --- | --- | --- | --- | --- | --- | --- | --- | --- | --- | --- | --- | --- | --- |
| 42 | 62.679% | 73.274% | 69.673% | 73.146% | 75.353% | 78.024% | 73.936% | 76.193% | 78.186% | 78.071% | 77.166% | 80.580% | 80.673% | 78.869% | 81.510% |
| 43 | 67.735% | 71.066% | 70.341% | 72.907% | 75.861% | 77.572% | 75.185% | 76.003% | 79.403% | 78.653% | 77.137% | 80.062% | 81.403% | 78.570% | 81.935% |
| 44 | 65.370% | 68.816% | 67.554% | 72.730% | 74.965% | 75.129% | 74.601% | 77.078% | 78.970% | 79.759% | 77.156% | 80.273% | 80.404% | 78.703% | 81.730% |
| 45 | 65.362% | 71.494% | 67.241% | 69.073% | 75.307% | 75.542% | 73.852% | 76.175% | 77.589% | 79.260% | 77.682% | 80.947% | 80.449% | 78.616% | 81.285% |
| 46 | 65.459% | 73.162% | 68.765% | 71.061% | 76.275% | 75.370% | 75.689% | 75.881% | 77.778% | 79.846% | 76.957% | 81.831% | 79.118% | 80.832% | 81.785% |

## Table S.8: AUROC results for LVEF classification using 1M ECG dataset (TemporalNet)

| **Seed** | **446 ECGs Baseline** | **446 ECGs Frozen** | **446 ECGs Finetuned** | **2,238 ECGs Baseline** | **2,238 ECGs Frozen** | **2,238 ECGs Finetuned** | **4,476 ECGs Baseline** | **4,476 ECGs Frozen** | **4,476 ECGs Finetuned** | **22,380 ECGs Baseline** | **22,380 ECGs Frozen** | **22,380 ECGs Finetuned** | **44,762 ECGs Baseline** | **44,762 ECGs Frozen** | **44,762 ECGs Finetuned** |
| --- | --- | --- | --- | --- | --- | --- | --- | --- | --- | --- | --- | --- | --- | --- | --- |
| 42 | 84.54% | 87.72% | 88.76% | 89.63% | 90.52% | 89.31% | 91.80% | 90.40% | 92.56% | 94.16% | 92.67% | 92.52% | 93.46% | 92.69% | 94.04% |
| 43 | 85.35% | 85.62% | 85.99% | 87.69% | 89.06% | 90.05% | 89.68% | 90.45% | 91.58% | 92.42% | 92.20% | 93.06% | 92.73% | 94.07% | 94.07% |
| 44 | 83.64% | 84.33% | 84.34% | 87.97% | 89.60% | 90.07% | 90.39% | 91.66% | 92.17% | 93.69% | 94.08% | 93.74% | 93.06% | 92.62% | 92.82% |
| 45 | 86.97% | 86.41% | 87.42% | 89.88% | 88.82% | 91.36% | 91.01% | 91.27% | 92.68% | 93.30% | 92.47% | 92.94% | 92.73% | 94.13% | 94.18% |
| 46 | 86.51% | 87.33% | 87.49% | 88.61% | 90.95% | 91.38% | 90.36% | 90.72% | 91.72% | 92.60% | 92.18% | 93.67% | 92.97% | 92.71% | 94.30% |

## Table S.9: AUROC results for KCL classification using 1M ECG dataset (TemporalNet)

| **Seed** | **523+47 Baseline** | **523+47 Frozen** | **523+47 Finetuned** | **2,618+237 Baseline** | **2,618+237 Frozen** | **2,618+237 Finetuned** | **5,236+474 Baseline** | **5,236+474 Frozen** | **5,236+474 Finetuned** | **26,183+2,371 Baseline** | **26,183+2,371 Frozen** | **26,183+2,371 Finetuned** | **52,367+4,742 Baseline** | **52,367+4,742 Frozen** | **52,367+4,742 Finetuned** |
| --- | --- | --- | --- | --- | --- | --- | --- | --- | --- | --- | --- | --- | --- | --- | --- |
| 42 | 66.19% | 72.13% | 71.32% | 74.36% | 75.95% | 77.33% | 78.50% | 78.94% | 78.33% | 81.03% | 81.73% | 79.91% | 82.59% | 80.35% | 82.53% |
| 43 | 68.89% | 74.43% | 74.03% | 74.76% | 77.67% | 78.18% | 77.49% | 78.26% | 79.42% | 81.37% | 79.97% | 81.01% | 82.48% | 80.19% | 82.25% |
| 44 | 70.21% | 70.99% | 64.75% | 74.17% | 75.91% | 76.49% | 77.22% | 78.55% | 78.28% | 81.19% | 79.67% | 81.31% | 82.72% | 80.10% | 83.48% |
| 45 | 64.04% | 72.51% | 72.19% | 73.84% | 77.19% | 76.97% | 78.20% | 79.15% | 78.21% | 81.37% | 80.14% | 81.36% | 82.54% | 80.47% | 82.46% |
| 46 | 65.31% | 73.65% | 71.62% | 74.36% | 76.91% | 77.68% | 77.32% | 78.47% | 78.86% | 81.07% | 79.80% | 80.64% | 82.41% | 80.49% | 82.43% |

## Table S.10: AUROC results for LVEF classification using 1M ECG dataset (Grouped Leads TemporalNet)

| **Seed** | **446 ECGs Baseline** | **446 ECGs Frozen** | **446 ECGs Finetuned** | **2,238 ECGs Baseline** | **2,238 ECGs Frozen** | **2,238 ECGs Finetuned** | **4,476 ECGs Baseline** | **4,476 ECGs Frozen** | **4,476 ECGs Finetuned** | **22,380 ECGs Baseline** | **22,380 ECGs Frozen** | **22,380 ECGs Finetuned** | **44,762 ECGs Baseline** | **44,762 ECGs Frozen** | **44,762 ECGs Finetuned** |
| --- | --- | --- | --- | --- | --- | --- | --- | --- | --- | --- | --- | --- | --- | --- | --- |
| 42 | 86.53% | 89.83% | 90.68% | 89.42% | 90.69% | 91.04% | 93.03% | 92.27% | 92.49% | 92.83% | 93.75% | 93.81% | 92.64% | 94.07% | 94.35% |
| 43 | 83.55% | 84.26% | 86.61% | 85.93% | 87.64% | 85.94% | 88.85% | 88.20% | 91.07% | 90.66% | 91.67% | 90.80% | 92.48% | 91.67% | 90.55% |
| 44 | 90.92% | 92.52% | 93.11% | 92.22% | 93.01% | 93.65% | 94.20% | 94.21% | 93.81% | 93.19% | 92.83% | 93.94% | 94.05% | 94.38% | 94.10% |
| 45 | 87.20% | 87.17% | 89.55% | 89.99% | 89.85% | 89.69% | 90.46% | 90.27% | 91.27% | 91.54% | 92.59% | 92.76% | 91.96% | 90.75% | 93.27% |
| 46 | 90.62% | 91.52% | 92.11% | 92.37% | 92.31% | 93.34% | 94.22% | 93.63% | 93.31% | 93.17% | 92.78% | 94.02% | 94.00% | 94.37% | 94.25% |

## Table S.11: AUROC results for KCL classification using 1M ECG dataset (Grouped Leads TemporalNet)

| **Seed** | **523+47 Baseline** | **523+47 Frozen** | **523+47 Finetuned** | **2,618+237 Baseline** | **2,618+237 Frozen** | **2,618+237 Finetuned** | **5,236+474 Baseline** | **5,236+474 Frozen** | **5,236+474 Finetuned** | **26,183+2,371 Baseline** | **26,183+2,371 Frozen** | **26,183+2,371 Finetuned** | **52,367+4,742 Baseline** | **52,367+4,742 Frozen** | **52,367+4,742 Finetuned** |
| --- | --- | --- | --- | --- | --- | --- | --- | --- | --- | --- | --- | --- | --- | --- | --- |
| 42 | 69.632% | 75.954% | 76.658% | 76.974% | 80.283% | 81.411% | 77.977% | 81.414% | 82.413% | 81.932% | 83.006% | 84.547% | 83.813% | 83.546% | 85.094% |
| 43 | 70.284% | 74.981% | 74.872% | 73.810% | 78.588% | 79.724% | 76.738% | 79.690% | 81.130% | 79.639% | 81.394% | 82.692% | 81.906% | 81.715% | 83.303% |
| 44 | 69.276% | 74.855% | 74.004% | 77.460% | 82.509% | 82.396% | 78.051% | 82.210% | 83.346% | 82.110% | 83.865% | 85.118% | 83.817% | 84.495% | 86.169% |
| 45 | 70.990% | 77.211% | 77.069% | 75.569% | 81.063% | 82.445% | 80.321% | 81.911% | 84.279% | 81.291% | 83.421% | 84.539% | 83.201% | 82.582% | 85.444% |
| 46 | 65.431% | 74.451% | 73.237% | 70.329% | 76.774% | 76.586% | 76.401% | 78.372% | 79.927% | 79.661% | 80.661% | 82.170% | 80.341% | 80.838% | 81.966% |
